# Structural basis of resistance to herbicides that target acetohydroxyacid synthase

**DOI:** 10.1038/s41467-022-31023-x

**Published:** 2022-06-11

**Authors:** Thierry Lonhienne, Yan Cheng, Mario D. Garcia, Shu Hong Hu, Yu Shang Low, Gerhard Schenk, Craig M. Williams, Luke W. Guddat

**Affiliations:** 1 School of Chemistry and Molecular Biosciences, The University of Queensland, Brisbane, QLD 4072 Australia; 2 Faculty of Food Science, Engineering and Biotechnology, Technical University of Ambato, Tungurahua, Ambato, 180210 Ecuador

**Keywords:** X-ray crystallography, Ligases, X-ray crystallography

## Abstract

Acetohydroxyacid synthase (AHAS) is the target for more than 50 commercial herbicides; first applied to crops in the 1980s. Since then, 197 site-of-action resistance isolates have been identified in weeds, with mutations at P197 and W574 the most prevalent. Consequently, AHAS is at risk of not being a useful target for crop protection. To develop new herbicides, a functional understanding to explain the effect these mutations have on activity is required. Here, we show that these mutations can have two effects (i) to reduce binding affinity of the herbicides and (ii) to abolish time-dependent accumulative inhibition, critical to the exceptional effectiveness of this class of herbicide. In the two mutants, conformational changes occur resulting in a loss of accumulative inhibition by most herbicides. However, bispyribac, a bulky herbicide is able to counteract the detrimental effects of these mutations, explaining why no site-of-action resistance has yet been reported for this herbicide.

## Abstract (web summary)

Acetohydroxyacid synthase (AHAS) is the target of more than 50 commercial herbicides, with many site-of-action resistance isolates identified in weeds. Here, the authors report the structural and kinetic characterizations to explain the effect AHAS mutations have on herbicide potency.

## Introduction

**Fig. 1:**
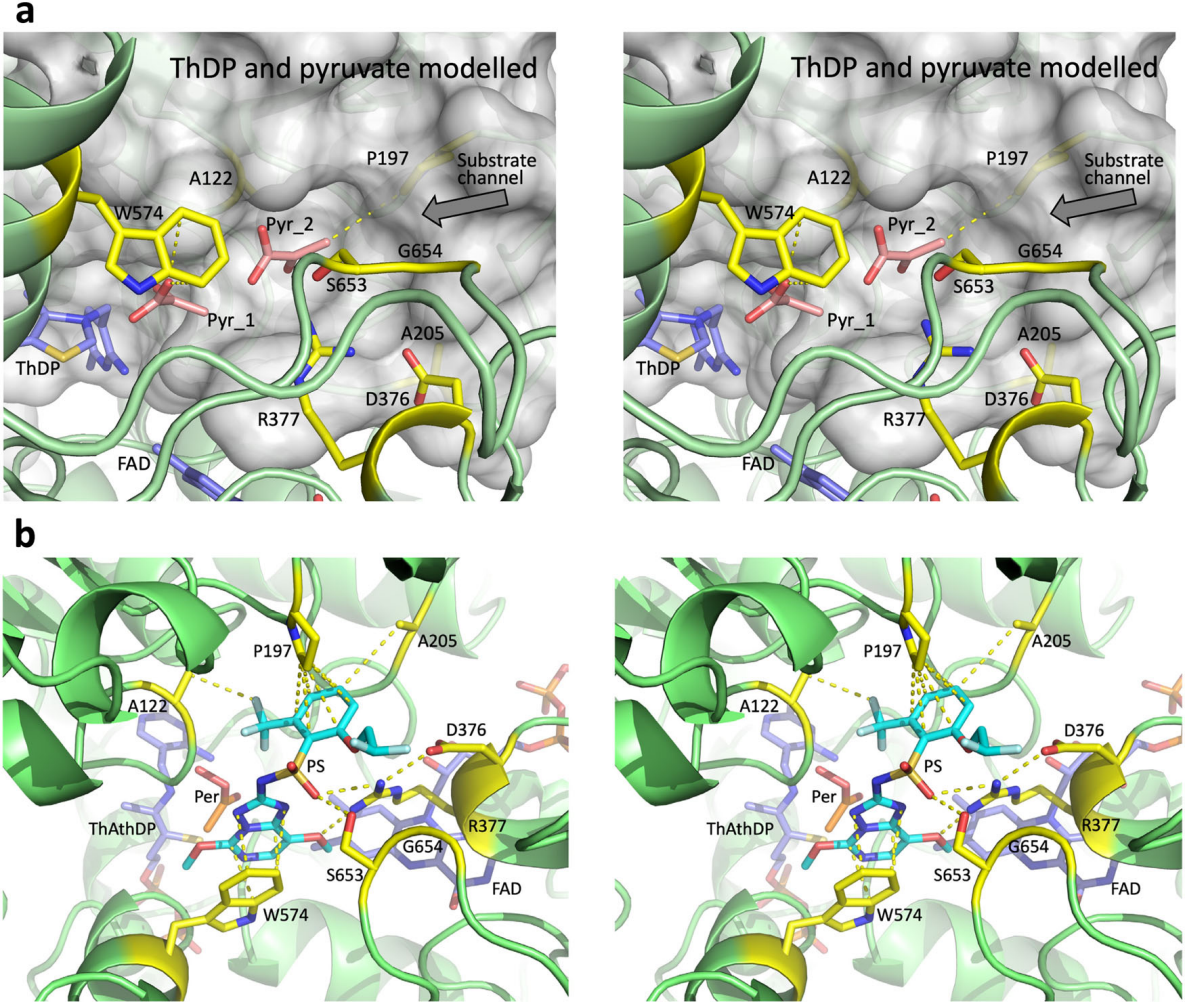
The location of the herbicide resistance sites in *Arabidopsis thaliana* AHAS (*At*AHAS). **a** Stereoview of *At*AHAS (PDB code: 5K6Q^30^) with pyruvate (Pyr_1 and Pyr_2) and an intact ThDP cofactor modeled. This model is based on the structure of the highly homologous *Saccharomyces cerevisiae* AHAS (*Sc*AHAS), whose structure has been solved in complex with pyruvate (PDB code: 6BD9^37^). The first pyruvate interacts with W574 through an anion–π interaction. The second pyruvate makes contact with P197. Residues that confer resistance (in yellow) upon herbicide selection pressure are found in the substrate access channel. **b** Stereoview of the *At*AHAS-penoxsulam structure (PDB code: 5WJ1,^11^) showing the positions of the residues involved in resistance relative to the herbicide, penoxsulam (PS, in cyan). “ThAthDP” and “Per” indicate thiamine thiazolone diphosphate (an oxidized product of ThDP) and peracetate, respectively.

Herbicides have been a mainstay in agriculture to protect crops against weed infestations for more than 70 years. However, across all the different classes of herbicides, resistance is becoming more prevalent, accelerating the need to discover new active compounds to replace those that are now of limited use. Acetohydroxyacid synthase (AHAS), also known as acetolactate synthase, is the first enzyme of the branched chain amino acid (BCAA) biosynthesis pathway, and is the target for more than 50 of these commercial herbicides^1,2^. These compounds fall into six different chemical classes, sulfonylureas (SUs), imidazolinones (IMIs), triazolopyrimidines (TPs), pyrimidinylbenzoates (PYBs), sulfonanilides and sulfonylamino-carbonyl-triazolinones (SCTs). The SUs and IMIs were first used commercially in the early 1980s^3–6^, and subsequently, the TPs, PYBs, sulfonanilides, and SCTs have been added to the market^7^. Since the introduction of these herbicides, 197 site-of-action resistance isolates have been identified. These sites are mainly focused on just eight amino acids (Supplementary Table 1)^1^ with all located in the substrate access channel or the catalytic pocket of AHAS (Fig. 1a). All eight sites are also direct contact points with the herbicides (Fig. 1b).

To date, crystal structures for the catalytic subunit of *Arabidopsis thaliana* AHAS (*At*AHAS) in complex with 13 different herbicides have been determined^8^. These include at least one member of each of the five different chemical classes of AHAS-inhibiting herbicides. These structures, along with detailed kinetic analyses, have provided a molecular understanding as to how these different structural motifs exert their activity as AHAS inhibitors.

In addition to these data, a crystal structure has also previously been obtained for the complex between bensulfuron-methyl (BSM) and a fungal AHAS holoenzyme formed by the association of catalytic and regulatory subunits^9^. This structure shows that only very minor conformational changes occur at the herbicide binding site when compared to the structure of the complex of the *Sc*AHAS catalytic subunit alone with BSM^9^. This result is in accord with the fact that in all three published AHAS holoenzyme structures (fungal, *Arabidopsis thaliana*^9^, and *Escherichia coli*^10^) the regulatory subunit does not disrupt the location where the herbicides bind. Thus, the influence of these mutations on the inhibition of the holo enzyme is likely to be conserved in the presence or absence of the regulatory subunit.

**Fig. 2:**
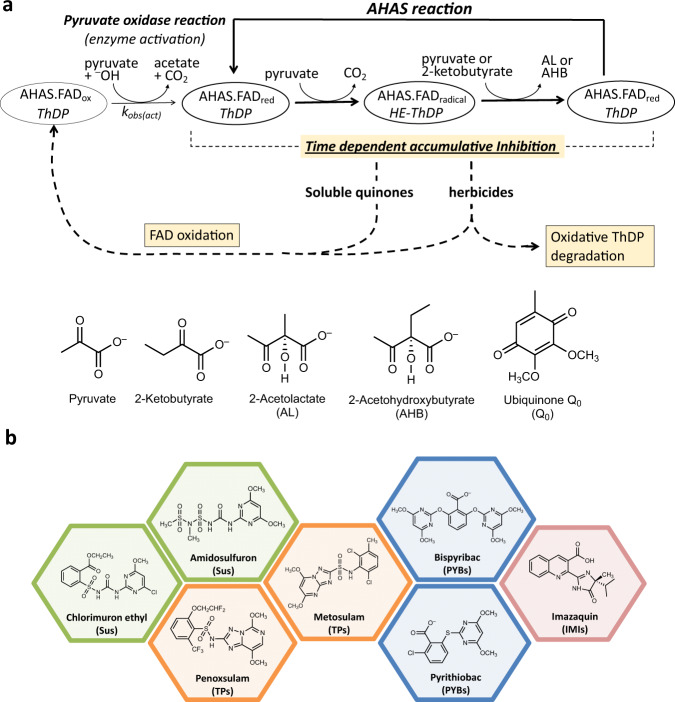
Reaction pathways, substrates, products, and inhibitors associated with the AHAS-catalyzed reaction. **a** Scheme representing the catalytic reaction of AHAS and its inhibition by soluble quinones and herbicides. **b** Chemical structures of the seven herbicides from four different classes studied here. SUs (chlorimuron ethyl (CE) and amidosulfuron (AS)), TPS (metosulam (MT) and penoxsulam (PS)), PYBs (bispyribac (BS) and pyrithiobac (PB) and IMIs (imazaquin (IQ)). FAD_ox_ = oxidized FAD. FAD_red_ = FAD reduced. FAD_rad_ = FAD semi-reduced. HE-ThDP = hydroxyethyl thiamine diphosphate.

Two factors contribute to the potency of the AHAS-inhibiting herbicides. One is the binding of the herbicide into the catalytic pocket, which blocks the substrate access channel^11^ and leads to the inhibition of the enzyme through substrate depletion (Fig. 1). The second involves the oxidative inactivation of the enzyme by the herbicide, in a process referred to as time-dependent accumulative inhibition^12^ (Fig. 2a). This mode of inhibition consists of a series of oxidative events in the active site that are triggered by the binding of the herbicide and involve the oxidation of the cofactors, ThDP, and FAD. These alterations induce the inactivation of the enzyme, which is prolonged after the inhibitor has left the catalytic pocket^11,12^. The potency of this type of inhibition does not always relate to the affinity of the herbicide to the enzyme. An example is imazaquin (IQ, an IMI) which has a relatively low affinity (*K*_*i*_ = 18 μM) for *At*AHAS but is, nonetheless, a potent herbicide. The potency for the IMIs thus stems from their ability to trigger a high level of accumulative inhibition, significantly more than the other classes of AHAS-inhibiting herbicides^13^.

The binding site for the AHAS-inhibiting herbicides is also associated with a downregulation pathway induced by redox signaling molecules, allowing BCAA synthesis to be attenuated where cells are lacking reducing power or are under oxidative stress^14^ (Fig. 2a). The mechanism of downregulation involves the oxidation of FAD by soluble quinone derivatives which in turn deactivates the enzyme. The re-reduction of FAD by the pyruvate oxidase (POX) side-reaction of AHAS^15^ is necessary to reactivate the enzyme, a process that is slow and accounts for the downregulation of AHAS activity^14^.

As mentioned above, the emergence of resistance is a major problem. To begin to overcome this problem, there have been several computational and experimental studies to design, screen, or discover new inhibitors of AHAS that could be developed as commercial herbicides^16–18^. All together, these studies have been successful in expanding the chemical space of inhibitors that can target AHAS resistance mutants. Amongst the most promising leads, there is a series of pyrimidine−biphenyl hybrids that show good herbicidal activity against AHAS inhibitor-resistant *Descurainia sophia* and *Ammannia arenaria*^19^. MB-QSAR (Mutation-dependent Biomacromolecular Quantitative Structure-Activity Relationships) is also a promising strategy for the discovery of new herbicides that could be effective against the on-set of resistance^18,20^. However, to date no experimentally determined structures are available for any herbicide in complex with AHAS mutants, making it difficult to explain the mechanisms of resistance, an important factor for consideration in the design of next generation AHAS-inhibiting herbicides.

In this work, the W574L, P197L, P197T, and S653T mutants have been selected for comprehensive kinetic analysis as they are the most commonly occurring site-of-action mutations in weeds. Crystal structures of the uninhibited W574L mutant, W574L mutant in complex with CE and BS, and the P197T mutant in complex with CE and BS have been solved. Together, the results reveal the mechanisms by which mutations alter the effectiveness of the commercial herbicides.

## Results

### Catalytic activity of the mutants

The *At*AHAS mutants, W574L, P197L, P197T, and S653T, were purified using the same procedure as the wild-type (WT) enzyme^13^. SDS-PAGE was used to assess the purity of the samples (Supplementary Fig. 1). Prior to investigating resistance mechanisms of the mutants, their catalytic efficiency (i.e.*, k*_cat_/*K*_*M*_) and ability to be regulated by redox signaling molecules were assessed. As *At*AHAS has a significant lag phase^14^, *K*_M_ and *k*_cat_ were determined by fitting the data to the implicit Michaelis–Menten equation (see “Methods” and Supplementary Figs. 2,  3).

The *K*_M_ value of pyruvate for WT *At*AHAS is 12.5 ± 0.6 mM (Supplementary Table 2), in good agreement with a previous study (11.6 mM^21^). The *K*_M_ values for the P197L, P197T, and S653T mutants (12.4, 16.5, and 15.5 mM, respectively) are comparable to the WT enzyme, suggesting the proline changes at position 197 and the change from serine to threonine at position 653 do not affect the environment for the binding of pyruvate. In contrast, the *K*_M_ value for the W574L mutant (48.3 mM) is around 4-fold higher compared to WT (Supplementary Table 2), in agreement with pyruvate directly interacting with W574 in *At*AHAS (Fig. 1a).

The *k*_cat_ for the P197L, P197T, and S653T mutants (6.3, 9.8, and 7.3 s^−1^, respectively) are similar to the WT enzyme (9.4 s^−1^), however, for the W574L mutant the *k*_cat_ is around 2.5-fold higher (25 s^−1^). The more rapid turnover number for the W574L mutant is likely due to a reduced number of interactions between substrate and enzyme in the pre-catalytic state (i.e., the decarboxylation of pyruvate) (Fig. 2a). The combined effect of changes in *k*_cat_ and *K*_*M*_ values is that the W574L mutation has only a small effect on the catalytic efficiency of *At*AHAS (Supplementary Table 2). This finding is in agreement with studies of other plant enzymes relying only a discontinuous assay, which may not accurately portray the kinetic profile for AHAS^22^.

### Regulation by redox signaling molecules

To start this analysis, we showed that the rate of the pyruvate oxidase side reaction (*k*_obs(act),_ see Fig. 2a^14^), which can be measured by monitoring the rate of activation of AHAS during the lag-phase of the enzyme^14^, is slightly lower in the mutants compared to WT (Supplementary Table 3 and Supplementary Fig. 4). Next, we investigated how the mutants responded to regulation by redox signaling molecules using ubiquinone Q_0_ (Q_0_) as an example^14^. The inhibition of *At*AHAS in the presence of 0.5 mM Q_0_ was shown to be slightly higher for the mutants compared to WT (Supplementary Table 3 and Supplementary Fig. 5), which correlates with the slower rate of recovery reflected by the lower *k*_obs(act)_ values. Overall, these variations are moderate, suggesting that regulation by redox signaling molecules is fully proficient in all the *At*AHAS mutants tested. These combined results show that the *At*AHAS mutants are fully competent, explaining why such mutations are tolerated in the weeds.

### Herbicide inhibition

The *K*_i_ values of the seven herbicides were measured for the four mutants (Table 1 and Supplementary Fig. 6). The data shows that the W574L mutation has a strong effect on all herbicides with *K*_*i*_ values increasing by at least 38-fold (IQ) and up to 35,000-fold (PS). This is consistent with the fact that W574 makes the strongest interactions with all the herbicides (see structural data below). The P197L, P197T, and S653T mutations also have a strong effect leading to an increase in *K*_*i*_ values, though generally not as much as compared with W574L. However, four mutants in combination with specific herbicides show a small decrease in *K*_*i*_ values compared to the WT enzyme (Table 1).

**Table 1:** *K*_*i*_ (nM) values of the seven commercial herbicides for WT and mutant *At*AHASs.

Herbicide (family)	WT	W574L	P197L	P197T	S653T
CE (SUs)	75 ± 6^a^	5200 ± 300	4400 ± 500	2200 ± 200	53 ± 4.6
AS (SUs)	4200 ± 600	870000 ± 60000	93500 ± 17100	61400 ± 8900	492 ± 25.0
PS (TPs)	1.9± 0.9^b^	67100 ± 3000	82 ± 7	85 ± 6.3	5 ± 2.2
MT (TPs)	29 ± 11.2	39300 ± 3900	1320 ± 130	1130 ± 130	66 ± 5.6
BS (PYBs)	41 ± 6^a^	6100 ± 1000	115 ± 13	106 ± 4.8	90 ± 13.1
PTB (PYBs)	179 ± 12^a^	91200 ± 8200	2000 ± 200	1500 ± 300	218 ± 15.8
IQ (IMIs)	18500 ± 2000^a^	704000 ± 58500	13800 ± 1100	12400 ± 1800	240000 ± 26000

*CE* chlorimuron ethyl, *AS* amidosulfuron, *PS* penoxsulam, *MT* metosulam, *BS* bispyribac, *PTB* pyrithiobac, *IQ* imazaquin.

^a^Taken from Garcia et al.^13^.

^b^Taken from Lonhienne et al.^11^.

“±” represents the standard error of the mean.

Time-dependent accumulative inhibition is also an important contributor to the potency of the AHAS-inhibiting herbicides (see “Introduction”). Therefore the apparent rate of enzyme inactivation (*k*_iapp_) and the rate of recovery (*k*_*3*_)^12^ for WT and mutant *At*AHAS in the presence of CE, AS, PS, BS, and IQ were measured. The results show that the W574L mutation abolishes accumulative inhibition for all inhibitors except for BS (Table 2 and Supplementary Figs. 7,  8). For the P197L/T mutants, the accumulative inhibition is abolished for all herbicides except BS and IQ. However, the S653T mutation has contrasting effects. The efficiency of accumulative inhibition (*k*_iapp_/*k*_*3*_) is increased for AS, PS, and BS but decreased for CE compared to WT (Table 2).

**Table 2:** Rate constants involved in accumulative inhibition (*k*_iapp_ and *k*_*3*_) for WT *At*AHAS and its mutants.

	WT	W574L	P197L	P197T	S653T
(CE) *k*_iapp_ (min^−1^)	1.24 ± 0.15	NAI	NAI	NAI	0.93 ± 0.1
(CE) *k*_*3*_ (min^−1^)	0.005 ± 0.001	NAI	NAI	NAI	0.015 ± 0.003
(CE) *k*_iapp_/*k*_*3*_	226	NAI	NAI	NAI	69
(AS) *k*_iapp_ (min^−1^)	0.39 ± 0.06	NAI	NAI	NAI	1.9 ± 0.01
(AS) *k*_*3*_ (min^−1^)	0.02 ± 0.001	NAI	NAI	NAI	0.02 ± 0.002
(AS) *k*_iapp_/*k*_*3*_	20	NAI	NAI	NAI	95
(PS) *k*_iapp_ (min^−1^)	1.22 ± 0.09	NAI	NAI	NAI	1.89 ± 0.12
(PS) *k*_*3*_ (min^−1^)	0.014 ± 0.002	NAI	NAI	NAI	0.015 ± 0.001
(PS) *k*_iapp_/*k*_*3*_	87	NAI	NAI	NAI	126
(BS) *k*_iapp_ (min^−1^)	0.32 ± 0.03	0.03 ± 0.006	1.15 ± 0.09	1.14 ± 0.11	0.66 ± 0.04
(BS) *k*_*3*_ (min^−1^)	0.018 ± 0.002	<1 × 10^−12^	0.037 ± 0.003	0.031 ± 0.004	0.017 ± 0.001
(BS) *k*_iapp_/*k*_*3*_	18	>10^11^	31	37	39
(IQ) *k*_iapp_ (min^−1^)	0.86 ± 0.03	NAI	0.26 ± 0.008	0.27 ± 0.012	0.37 ± 0.017
(IQ) *k*_*3*_ (min^−1^)	<1 × 10^−12^	NAI	<1 × 10^−12^	<1 × 10^−12^	<1 × 10^−12^
(IQ) *k*_iapp_/*k*_*3*_	>10^11^	NAI	>10^11^	>10^11^	>10^11^

*NAI* no accumulative inhibition detected.

“±” represents the standard error of the mean.

These results show that BS is an exception in that there is improved accumulative inhibition for the mutants compared to WT *At*AHAS, even tending towards being virtually irreversible (Table 2). To ascertain the potential of BS as an herbicide tolerant to resistance mutations, it is worth comparing the kinetics of inhibition (*K*_*i*_ and accumulative inhibition) of BS for W574L with IQ for WT. The *K*_*i*_ of BS for the W574L mutant is 6.1 μM, a lower value than for IQ in the WT (18.5 μM, Table 1). The accumulative inhibition is irreversible for both BS in the W574L mutant and IQ in WT (Table 2). Thus, as IQ is a potent inhibitor of weed growth, despite having a high *K*_*i*_, it is a reasonable expectation that BS also be an effective inhibitor of weed growth, even when a weed possesses the W574L mutation. BS also shows only a moderate loss of affinity (~50%) for the P197L/T and S653T mutants; however, with slightly improved accumulative inhibition. Thus, it is apparent that this herbicide is significantly less impacted by these mutations compared with the other AHAS-inhibiting herbicides.

The in vitro inhibition of the mutants by the herbicides evaluated here correlates with the experimental observations made for PTBs, IMIs, Sus, and TPs in the field^1^ (Supplementary Table 4). Currently, there is no field data for the inhibition of the mutant weeds by PYBs (Fig. 2b) suggesting such changes are rare or simply do not occur. Of the 38 W574L mutants reported in weeds, 37 are fully resistant and one is partially resistant to the four classes of herbicides. In agreement with previous observations, findings herein show that W574L strongly reduces the binding affinity of the herbicide families evaluated, and abolishes their ability to engender accumulative inhibition (Tables 1 and 2). In the P197L/T mutants, CE (SU), AS (SU), and PS (TP) are negatively impacted in their ability to inhibit the enzyme, whereas IQ (IMI) is an exception with inhibitory activity increasing. Accordingly, amongst all herbicide families, the IMIs are the inhibitors that are least affected by the P197L/T mutations in the field (Supplementary Table 4). Furthermore, the presented data also demonstrated that the S653T mutant only has a small influence on the inhibition potency of the majority of herbicides, except for IQ where the *K*_*i*_ increases by more than 10-fold (Table 1). The field data obtained confirm this result as all S653T weed mutants are resistant to the IMIs. This is in contrast to the other families where the mutation has almost no effect (Supplementary Table 4).

### Crystal structures of the *At*AHAS mutants in complex with two commercial herbicides

To determine how the resistance mutations affect the mode of binding of the inhibitors five crystal structures were determined (Supplementary Table 5).

Firstly, the structure of the W574L *At*AHAS mutant was determined showing it has an almost identical fold with the WT enzyme, with a rmsd value of 0.146 Å after superimposition of 557 out of 582 Cα atoms. Crucially, no structural change in the environment of W574L other than the replacement of the side-chain is observed (Supplementary Fig. 9). Thus, the differences in kinetic properties can be attributed to the mutation and not to any additional conformational change in the protein structure.

**Fig. 3:**
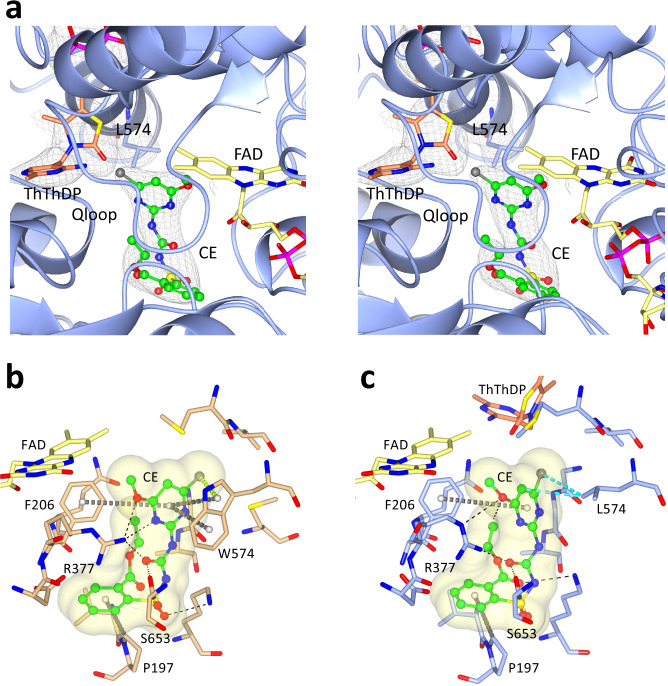
Binding mode of CE in WT *At*AHAS and in the W574L mutant. **a** Stereoview of the CE binding site in W574L. ThDP has been oxidized to thiamine thiazolone diphosphate (ThThDP, light brown). Electron densities (2F_o_-F_c_ map) in light brown correspond to CE, ThThDP and L574 contoured at 1, 2, and 1σ, respectively. **b** Interactions between CE and WT *At*AHAS (PDB code: 1YBH^38^). In this structure, ThDP is almost completely degraded and therefore not observed in the density map. **c** Interactions between CE and the W574L mutant. (All panels) CE is green and in ball and stick representation. FAD is yellow. Dashed lines represent hydrogen bonds (black). The grey dashed cylinders represent π–π and carbon–π interactions. The lime dashed cylinders represent halogen–π interactions. The cyan dashed cylinders represent hydrophobic interactions. Q-loop is a loop in the active site that includes residues 193 to 210. *ThThDP* thiamine thiazolone diphosphate.

Next, the crystal structure of the W574L mutant in complex with CE was determined. CE is a member of the SU family (Fig. 2b). A co-crystal structure in complex with WT AHAS has previously been determined to 2.5 Å (PDB code: 1YBH), but herein the structure of the W574L mutant-CE complex is presented. Although the resolution is lower than for WT, the complete electron density for CE and surrounding environment is unequivocal (Fig. 3a). The location of CE in the herbicide binding site in the mutant is similar to that when it binds to WT, but some changes in conformation are observed (Fig. 3b). When leucine replaces tryptophan, there is a dramatic decrease in the number of enzyme-inhibitor interactions. In the WT complex, the pyrimidine makes π–π interactions and a halogen–π interaction with the indole moiety of W574. In the mutant complex, those interactions are replaced by a hydrophobic interaction between a methyl group of the mutated L574 and the pyrimidine ring of CE. This change is the main factor accounting for the 70-fold loss of affinity of CE for this mutant compared to the WT enzyme (Table 1).

**Fig. 4:**
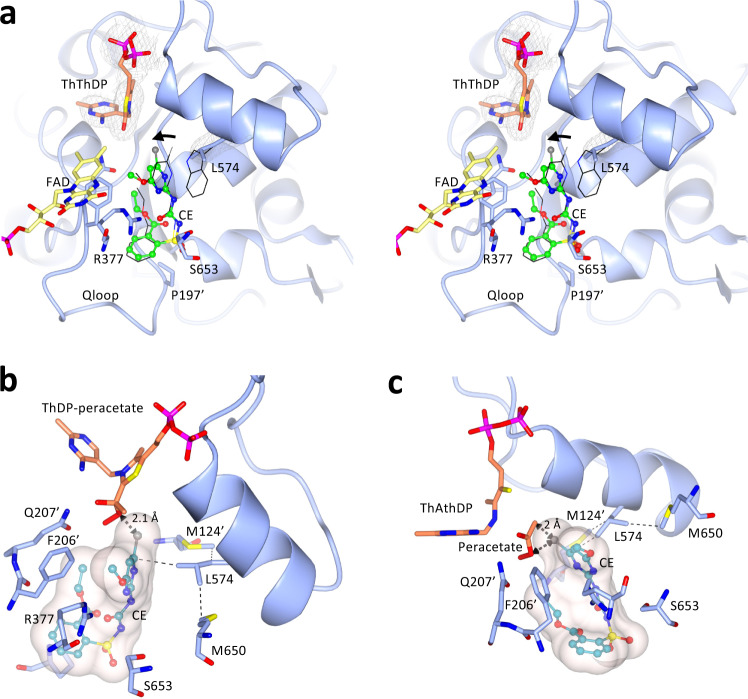
Conformational changes to CE that are induced by the W574L mutation. **a** Stereo view of the herbicide binding site in the W574L:CE complex. The AHAS polypeptide is in light blue. FAD, ThThDP, and CE are in yellow, light brown, and green carbon atoms, respectively. The black thin lines represent CE and W574 in the *At*AHAS-CE structure that has been superposed to the W574L-CE structure. The black arrow indicates the rotation of the pyrimidine ring of CE. The 2F_o_-F_c_ electron density map is in gray mesh. The position of CE relative to (**b**) ThDP-peracetate and (**c**) ThAthDP and peracetate as they appear in the *Sc*AHAS-PS (PDB code: 5WKC^11^) complex superimposed with the protein and herbicide coordinates of the W574L-CE structure. Thin dashed lines represent hydrophobic interactions. *ThAthDP* thiamineaminoethenethiol diphosphate.

In comparing the structures of the WT and W574L complexes, the most striking difference is a rotation of 38° in the dihedral angle that connects the heterocyclic ring of CE to the sulfonylurea bridge (Fig. 4a). For the chlorine atom to make an optimal hydrophobic interaction with the side chain of leucine (Fig. 3c), it is forced towards the region where ThDP is located. Note that in this structure, ThDP is replaced by thiamine thiazolone diphosphate (ThThDP), an oxidized derivative of ThDP. Such a derivative has been observed in many other AHAS structures in complex with various herbicides^13^. Importantly, the position adopted by the chlorine atom in the W574L mutant has the potential to impede accumulative inhibition imposed by CE. Accumulative inhibition involves two major steps: i) the formation of ThDP-peracetate promoted by the binding of the herbicide^11^, and ii) the fragmentation of ThDP-peracetate into thiamine aminoethenethiol diphosphate (ThAthDP) and free peracetate that acts as reactive oxygen species able to oxidize FAD and possibly several methionine side-chains near the active site^11,13^. The superimposition of the structure of the W574L-CE complex with the structure of *Saccharomyces cerevisiae* (*Sc*) AHAS in complex with the herbicide, penoxsulam (PDB code: 5WKC^11^) shows that the chlorine atom of CE would sterically clash with the peracetate moiety of ThDP-peracetate (the distance between them is only 2.1 Å) (Fig. 4b) or with the free peracetate (with a distance between them of only 2 Å) (Fig. 4c), thus abolishing accumulative inhibition (Table 2).

**Fig. 5:**
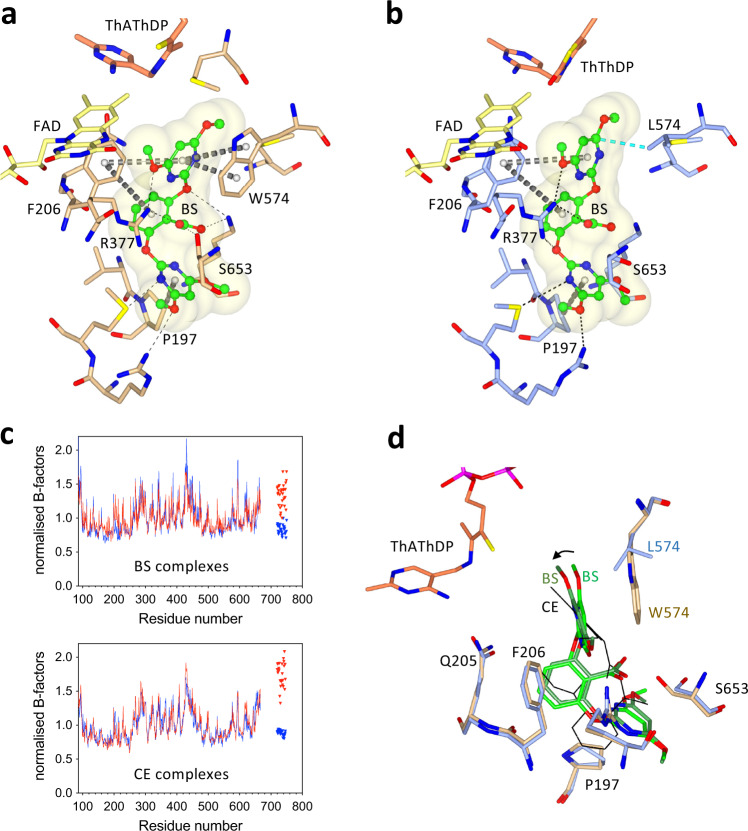
The binding mode of BS in WT and in the W574L *At*AHAS mutant. **a** The interactions of BS with WT *At*AHAS (PDB code: 1YBH^38^). ThDP is degraded to ThAThDP (light brown). **b** The interactions of BS with the W574L mutant. ThDP is oxidized to ThThDP (light brown). **a**, **b** BS is in green and ball and stick representation. The FAD is in yellow and in stick representation. Dashed lines represent hydrogen bonds (black). Grey dashed cylinders represent π–π interactions. Blue dashed cylinders represent hydrophobic interactions. **c** B-factor plot for the polypeptide and herbicide. An increase in normalized B-factor for the herbicide indicates it is not bound as tightly to the mutant as it is to the WT enzyme (top panel). However, the destabilization is not as profound compared (lower panel) to the normalized B-factors for the two CE structures (i.e., WT and W574L mutants). Blue and red represent WT and mutant enzymes, respectively. Lines and triangles represent polypeptide and inhibitor, respectively. **d** Superimposition of the WT *At*AHAS-BS, W574L-BS, and W574L-CE complexes. The superimposition of the WT *At*AHAS-BS and W574L-BS structures shows the rotation of the heterocyclic ring that occurs when W574 is mutated to leucine. The superimposition with W574L-CE (CE represented by thin black sticks) shows that the movement of the heterocyclic ring in the W574L mutant is greater for CE than BS.

Next, the crystal structure of the W574L mutant in complex with BS was determined, showing the overall position of BS is unchanged compared to WT *At*AHAS (Fig. 5a, b). As for CE, the structure revealed that the binding of BS is strongly affected by the loss of π−π interactions when leucine replaces tryptophan (Fig. 5b). This correlates with the loss of affinity (150-fold, Table 1), this despite the fact that the two other aromatic rings contained within BS make numerous conserved interactions with the polypeptide (Fig. 5a, b) the observation that there is only a small increase in B-factors for BS when compared to its binding with the WT enzyme (Fig. 5c). In summary, the increased complexity of BS, and inherent lack in degrees of rotational freedom, reduces the ability of this herbicide to compensate for steric clashes with L574.

As for CE, the presence of leucine triggers the rotation of the heterocyclic ring, however, to a lesser extent (Fig. 5d). This variation is likely related to the fact that ThAThDP is observed in the *At*AHAS-BS structure, in contrast to the *At*AHAS-CE structure where only ThThDP is observed (Fig. 4a). Indeed, refinements of the structure with various products (ThThDP vs ThAThDP plus peracetate) suggest that ThDP in this structure is a mixture of ThThDP and ThAThDP/peracetate (Supplementary Fig. 10).

**Fig. 6:**
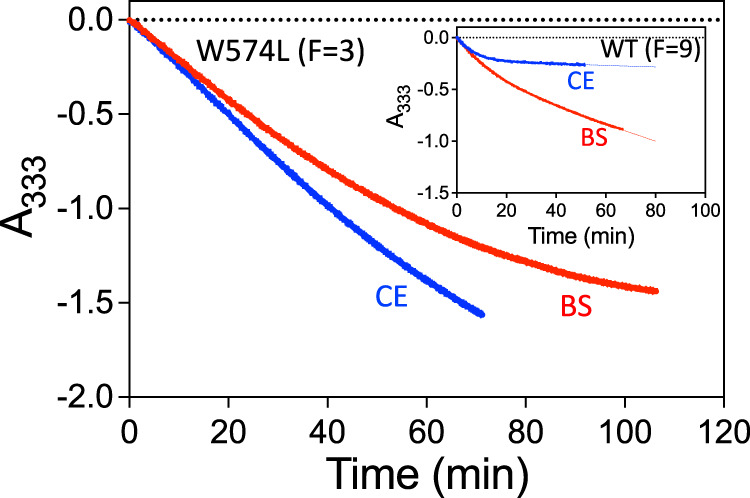
Progress curves for the accumulative inhibition of W574L and WT *At*AHAS by CE and BS. In WT *At*AHAS (inset), CE promotes a stronger level of accumulative inhibition than BS. However, in the W574L mutant, the presence of CE accumulative inhibition is virtually abolished. In contrast, BS still can promote accumulative inhibition in the W574L mutant. F = the ratio of enzyme concentration vs inhibitor concentration. Source data are provided with the paper.

These structural observations suggest that in contrast to CE, when BS binds to the W574L mutant it is possible for ThAthDP and free peracetate to co-exist with the inhibitor. In agreement with this, the kinetic data show BS can induce accumulative inhibition in the W574L mutant (Fig. 6). The ability of CE to promote accumulative inhibition in the W574L mutant has been virtually abolished relative to WT. In contrast, the mutation does not abolish the accumulative inhibition induced by BS (Fig. 6). The combined kinetic and structural data thus emphasize that small conformational changes in the vicinity of the mutated residue can lead to significant differences in the level of accumulative inhibition, in correlation with the amount of space available for the generation of oxidative species.

**Fig. 7:**
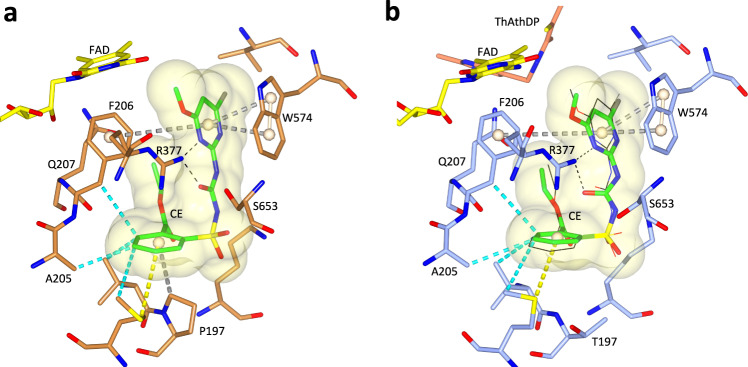
The binding mode of CE in WT *At*AHAS and in the P197T mutant. **a** The interactions of CE with WT *At*AHAS (PDB code: 5K3S^13^). **b** The interactions of CE with the P197T mutant. ThDP is degraded to ThAThDP (light brown). The black thin lines represent CE from the WT *At*AHAS:CE structure that has been superimposed with the P197T-CE structure. (**a**, **b**) CE is in green and in ball and stick representation. The FAD is in yellow and in stick representation. Dashed lines represent hydrogen bonds (black). Grey dashed cylinders represent π–π interactions. Yellow dashed cylinders represent sulfur–π interactions. Blue dashed cylinders represent hydrophobic interactions. Pale brown spheres represent the geometric centers of the aromatic groups.

We then determined the crystal structure of the P197T mutant in complex with CE. Overall, the structure of the polypeptide of the P197T-CE complex is similar to the structure of the WT *At*AHAS-CE complex (rmsd of 0.206 Å for all Ca atoms). In addition, when CE is in complex with the mutant the majority of interactions are conserved (Fig. 7). This suggests that the 30-fold loss of affinity is due to the loss of interactions between the aromatic ring of CE and the pyrrolidine ring of P197. This is consistent with a study that demonstrated the stabilization energy between a proline residue and an aromatic ring moiety of a ligand can range from 8 to 25 kJ/mol, depending on the relative positions of the pyrrolidine ring and the aromatic system^23^. Here, by applying the Gibbs free energy formula (*∆G* = −*R.T*.ln*K*), the loss of stabilization energy is calculated to be 8.5 kJ/mol.

The kinetic data highlights that the P197T and P197L mutations abolish accumulative inhibition invoked by CE (Table 2). The structure itself does not provide a conclusive explanation for the loss of accumulative inhibition by CE in these mutants, but in *Sc*AHAS, the proline residue at the equivalent position has been proposed to play a major role in the rate of enzyme inactivation (*k*_iapp_)^11^. This feature is therefore likely to be responsible for the lack of accumulative inhibition by CE in the P197 mutants.

**Fig. 8:**
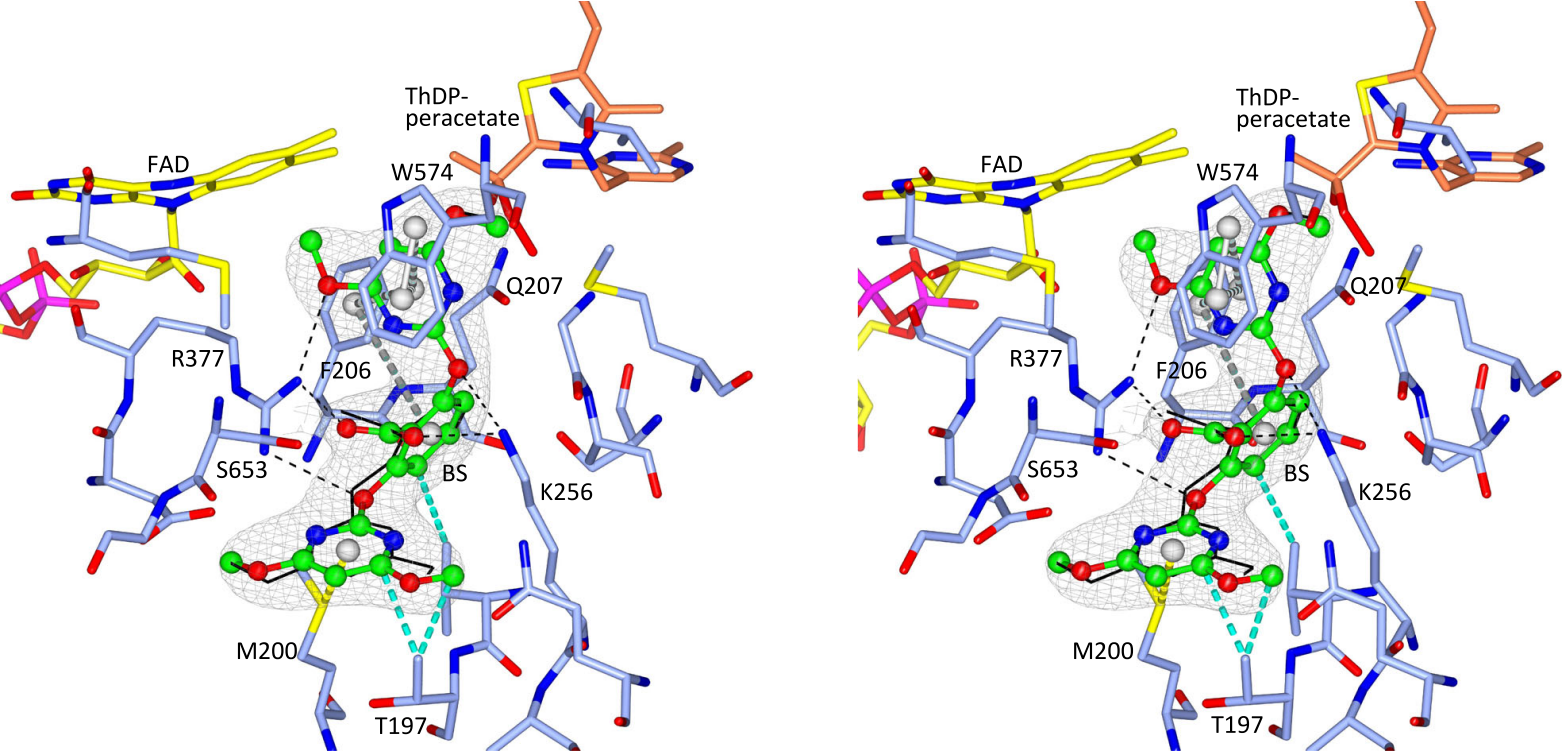
Stereoview of the herbicide binding site in the P197T-BS complex. The *At*AHAS polypeptide is in light blue. FAD, ThAThDP, and BS are in yellow, light brown, and green carbon atoms, respectively. The black thin lines represent BS in the WT *At*AHAS-BS structure that has been superimposed to the P197T-BS structure. Electron densities (2F_o_-F_c_ map) in gray correspond to BS contoured at 1.2 σ. The interaction representations are as in Fig. 7. Grey dashed cylinders represent π–π interactions. Yellow dashed cylinders represent sulfur–π interactions. Blue dashed cylinders represent hydrophobic interactions. Dashed lines represent hydrogen bonds (black).

Next, the crystal structure of the P197T mutant in complex with BS was determined. The P197T-BS complex is very similar to the structure of the WT *At*AHAS-BS complex, with the polypeptides and the herbicides having near-identical conformations (Fig. 8). In contrast to CE (Fig. 7), BS interacts with T197. This feature and the fact that the interaction of the two heterocyclic rings is not affected by the P197 mutation may explain why the binding of BS is only weakly affected, with an increase in *K*_*i*_ of around three-fold compared to WT *At*AHAS (Table 1). Remarkably, the efficiency of accumulative inhibition by BS increases in the P197 mutant by approximatively two-fold (Table 2). Altogether, these data suggest that the inhibition potency of BS is not affected by the P197 mutation.

## Discussion

This investigation highlights the importance of combining extensive kinetic measurements with detailed structural analyses to facilitate the accurate determination of the molecular basis for herbicide resistance. The process of resistance involving AHAS-inhibiting herbicides is much more complex than a result of a simple reduction in affinity for the enzyme due to mutations. As detailed herein, the process of accumulative inhibition due to an oxidative side reaction of AHAS plays an important role in the effectiveness of these herbicides. A major advance in explaining resistance is the importance of the available free space around ThDP to allow accumulative inhibition to occur. The comparison of structural movements of the herbicides BS and CE in the active sites of the W574L, P197T, and P197L mutants of *At*AHAS provides insight into factors that underpin the occurrence of accumulative inhibition. In the case of CE, accumulative inhibition was observed to be almost completely abolished for all three mutants. However, for BS accumulative inhibition was maintained for each mutant, and for the two P197 mutants the affinity of BS (*K*_i_) remained high. Overall, this study highlights BS as an efficient and robust herbicide, in particular against weeds that possess the most common mutations observed in current field isolates. The highly aromatic and flexible nature of BS (i.e., bisheteroaryl ether), whose structure is highly unusual compared to the other AHAS-inhibiting herbicides, appears to be a tactical advantage in combatting resistance caused by the introduction of the current mutations. This suggestion is in agreement with previous studies showing that flexibility and the addition of a third aromatic ring are properties that have the potential to combat resistance^24–27^. In rational structure-based drug/herbicide design the natural inclination is to construct compounds that can completely fill the target binding pocket. However, in the AHAS situation, it is essential that the space where the oxidative side-reaction occurs be left available for accumulative inhibition to occur. It is therefore suggested that future design efforts should be concentrated on inhibitors that avoid entering that space. In this regard, it is worth pointing out the mode of binding of the recently isolated natural product inhibitor of AHAS, harzianic acid^28^. This compound not only takes a conformation avoiding the region where the oxidative side-reaction occurs, but also makes interactions within a region of the catalytic pocket that has not been used by any of the existing commercial herbicides. Thus, it is a strong candidate for adaption or use as a next-generation herbicide.

The high-level of site-of-action resistance mutations for the AHAS-inhibiting herbicides that occurred in the 1980s, 1990s, and early 2000s was in part due to their over-use and without an awareness into the potential risks of the development of resistance. In more recent times, with improved stewardship, there has been a reduction in the number of site-of-action resistant weeds, but they do still occur. To further slow the emergence of resistance, strategies such as combination formulations could be employed here. Such a strategy could be especially useful where resistance mutations arise that lead to steric hindrance.

To conclude, the structural and detailed kinetic studies undertaken here provide insights to suggest that there is a need to maintain a strong level of accumulative inhibition when faced with site-of-action mutations. Future MB-QSAR studies that take into account the structures presented here and the role of accumulative inhibition are likely to be effective in the design of new herbicide leads with a reduced propensity for the further development of resistance.

## Methods

### Expression and purification of WT and mutant *At*AHAS

All of the constructs for the mutants were obtained using the QuickChange^®^ Site-Directed Mutagenesis kit (Agilent). The plasmid containing the His-tagged *At*AHAS wild-type enzyme^29^ was amplified by PCR using the *PfuUltra* polymerase with the primers shown in Supplementary Table 6. The parental plasmid was then digested with *Dpn* I restriction enzyme and the resulting mutant *At*AHAS containing constructs transformed into *E. coli* DH5α chemically competent cells. The correct introduction of each mutation was confirmed by Sanger sequencing at the Australian Genome Research Facility. For the expression and purification of WT and mutant *At*AHAS followed a previous protocol, but with some modifications^13,30^. The procedure applied here was to start with *E. coli* BL21 (DE3) transformed cells grown in Terrific Broth media at room temperature. When the OD_600_ reached 2.0, the expression of the protein was induced by 0.1 mM IPTG at 16 °C and 150 opm. The cell culture was then centrifuged at 4 °C, 3300 × *g* for 20 min, and pellets were collected and stored at −80 °C.

For lysis, the pellet was resuspended in an IMAC binding buffer (50 mM Tris, pH 7.9, 10 mM MgCl_2_, 1 mM ThDP, 10 μM FAD, and 20 mM imidazole) supplemented with 10 mg lysozyme per gram of cell, 50 μL DNase (50 μg/μL) and 1 mM PMSF. Sonication was achieved using 18% amplitude, 1 min cycle with 10 s intervals. The solution was then centrifuged at 39,000 × *g* for 40 min with the supernatant used for purification.

For IMAC purification, the elution buffer consisted of 50 mM Tris (pH 7.9), 10 mM MgCl_2_, 1 mM ThDP, 10 μM FAD and 300 mM imidazole. Subsequently, the protein peak was loaded onto a S-300 HR gel filtration column (Pharmacia) previously equilibrated with 50 mM KPO_4_ (pH 7.2), 10 mM MgCl_2_, 1 mM ThDP, 10 μM FAD, and 1 mM DTT. The protein was eluted with 400 mL of the same buffer and the flow through was collected in 5 mL fractions. The fractions containing the protein were pooled and concentered using an Amicon concentrator device (30 kDa MWCO). The purified enzymes were stored in 30 mL aliquots at −80 °C. Protein purity was assessed by SDS-PAGE (Supplementary Fig. 1).

### AHAS assays

Standard assays were performed using a continuous spectrophotometric method measuring the disappearance of pyruvate^31^. The standard buffer contained 50 mM potassium phosphate, pH 7.2, 1 mM ThDP, 10 mM MgCl_2_, and 10 µM FAD. The concentration of pyruvate was in the range 100–160 mM, as described in the text, methods, or figure legends.

For *K*_*M*_ and *k*_cat_ measurements, it was difficult to measure the initial velocities of fully activate enzyme due to the lag-phase (Supplementary Fig. 11), especially at low pyruvate concentrations where this lag-phase is augmented^14^. To overcome this problem, we measured the *K*_M_ and *k*_cat_ using the implicit Michaelis–Menten equation relating the substrate consumption to time. This was instead of the more usual equation relating initial velocities to the concentration of substrate, thus avoiding the extremely long lag phase at low pyruvate concentrations.

### Derivation of the implicit Michaelis–Menten equation

In the initial step, the conventional Michaelis Menten equation,

1 $$v=\frac{{v}_{{\max }}[S]}{{K}_{M}+[S]}$$

where *v/v*_max_ represents the velocity/maximum velocity of the reaction and [*S*] the concentration of substrate, is transformed as follows:

2 $$-\frac{1}{2}\frac{{{{{{\rm{d}}}}}}\left[S\right]}{{{{{{{\rm{d}}}}}}t}}=\frac{{v}_{{\max }}\left[S\right]}{{K}_{M}+\left[S\right]}$$

which can be rearranged to

3 $${K}_{M}\frac{{{{{{\rm{d}}}}}}\left[S\right]}{[S]}+{{{{{\rm{d}}}}}}\left[S\right]=-2{v}_{{\max }}{{{{{{\rm{d}}}}}}t}$$

Equations (2) and (3) take into account the fact that the product (2-acetolactate) is the result of the condensation of two pyruvate molecules.

Equation (3) can be integrated

4 $${\int }_{{S}_{0}}^{{S}_{t}}\left({K}_{M}/[S]\right){{{{{{\rm{d}}}}}}S}+{\int }_{{S}_{0}}^{{S}_{t}}{{{{{{\rm{d}}}}}}S}={\int }_{{t}_{0}}^{t}-2{v}_{{\max }}{{{{{{\rm{d}}}}}}t}$$

resulting in the following equation

5 $${S}_{t}-{S}_{{{{{{\rm{tot}}}}}}}=-2{v}_{{\max }}\left(t-{t}_{0}\right)-{K}_{M}\cdot {{{{{\rm{ln}}}}}}\frac{{S}_{t}}{{S}_{{{{{{\rm{tot}}}}}}}}$$

where *S*_*t*_ represents the concentration of substrate (given in absorbance units at 333 nm) at a given time (*t*) and *S*_tot_ the total concentration of substrate at the start of the reaction (*t*_*0*_). Note that t_0_ does not correspond to the actual start of the reaction, but instead to a designated time corresponding to the start of a reaction without lag-phase (Supplementary Fig. 8).

Taking into account that

6 $$y=({S}_{t}-{S}_{{{{{{\rm{tot}}}}}}})$$

where *y* is the absorbance reading at 333 nm during the reaction (Supplementary Fig. 8), Eq. (5) can be transformed as follows:

7 $$y=-2{v}_{{\max }}\left(t-{t}_{0}\right)-{K}_{M}\cdot {{{{{\rm{ln}}}}}}\left(1+\frac{y}{{S}_{{{{{{\rm{tot}}}}}}}}\right)$$

The data were then fitted to Eq. (7) using GraphPAD Prism V 9.3 and the script

8 $$Y=-2\cdot {VM}\cdot \left(X-{TO}\right)-{KM}\cdot {{{{{\rm{ln}}}}}}\left(1+\frac{Y}{S0}\right)$$

with the value of *S0* corresponding to *S*_tot_, the concentration of pyruvate at the start of the reaction. The resultant *K*_*M*_ value, given in absorbance units, is then divided by the molar extinction coefficient of pyruvate at 333 nm (ε333 = 17.5 M^−1^cm^−1^), to obtain a *K*_*M*_ value in *M*.

### AHAS activation and time-dependent accumulative inhibition

*At*AHAS was assayed in the standard assay buffer containing 160 mM pyruvate, at 30 °C. For *At*AHAS activation the progress curve corresponding to the lag-phase was fitted to Eq. (8) of Lonhienne et al.^14^. For the time dependent accumulative inhibition of *At*AHAS the progress curves were fitted to Equation 9 of Lonhienne et al.^12^.

### Crystallization and structure determination

The hanging-drop vapor-diffusion method was used to prepare the crystals. The well solution consisted of 0.1 M CHES (pH 9.4–9,8), 1 M Na/K tartrate, and 0.18–0.2 M (NH_4_)_2_SO_4_. In the drop, the ratio of protein and well solution was 2:1.

Beam line MX1 at the Australian Synchrotron was used to collect the X-ray data^32^. The data were scaled and merged using XDS^33^. All of the structures were solved by molecular replacement using the *At*AHAS-CE complex (pdb code: 1YBH) as a starting model and the program Phaser (in Phenix 1.9)^34^. Win-Coot 0.8.9^35^ was used to build the models for the structures. Structural dictionary files were made using Elbow in Phenix 1.9^36^. Omit electron density maps are shown in Supplementary Fig. 12.

### Reporting summary

Further information on research design is available in the Nature Research Reporting Summary linked to this article.

## Supplementary information

Supplementary Information

Reporting Summary

## Data Availability

All coordinates and structure factors have deposited in the protein data bank. The access codes are 7U1U, 7U25, 7STQ, 7U1D, and 7TZZ for the uninhibited W574L mutant, the W574L-BS complex, the W574L-CE complex, the P197L-CE complex, and the P197-BS complex, respectively. Constructs for wild type and the mutant AHASs are available upon request. Source data are provided with this paper.

## Source data

Source Data
