# Supplementary material for: Structural basis of resistance to herbicides that target acetohydroxyacid synthase
Source: Nat Commun. 2022 Jun 11;13:3368. doi: 10.1038/s41467-022-31023-x (PMC9188596; doi:10.1038/s41467-022-31023-x)
Supplement: Supplementary file 1 — Supplementary Information [file 41467_2022_31023_MOESM1_ESM.pdf]

Structural basis of resistance to herbicides that target acetohydroxyacid synthase

Lonhienne *et al.*

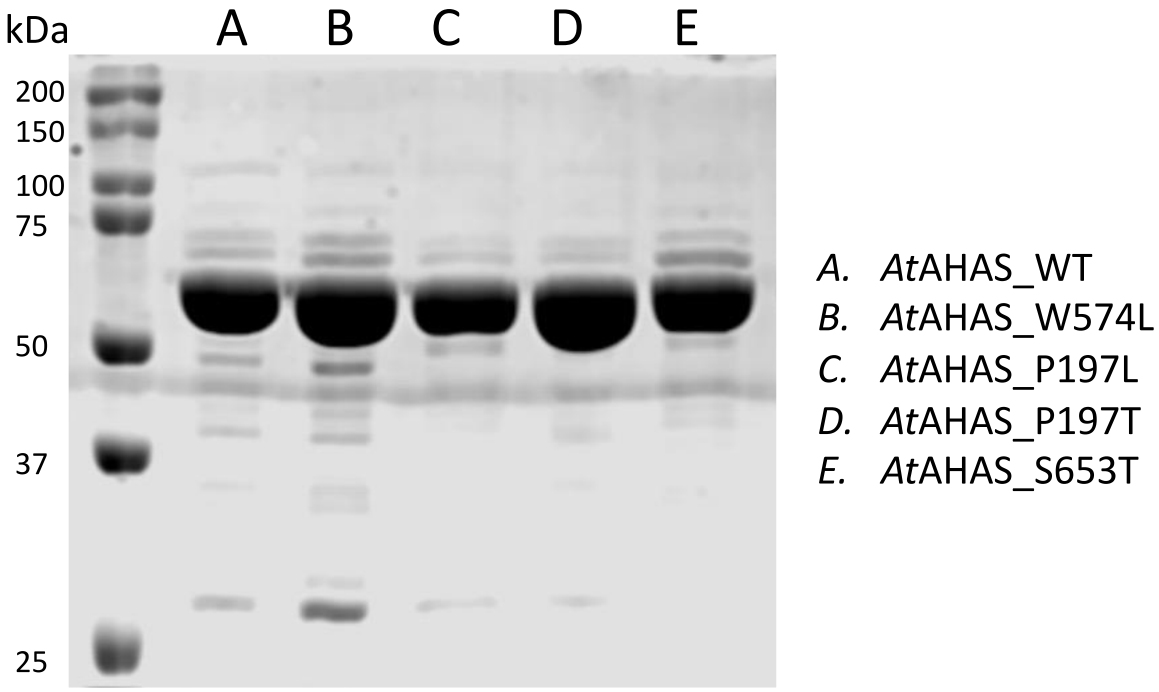

Supplementary Figure 1. **SDS-PAGE for purified WT *At*AHAS and the mutants.** Prior to investigating resistance mechanisms of the mutants, their catalytic efficiency (*i.e.* k_{cat}/K_M) and ability to be regulated by redox signalling molecules was assessed. As *At*AHAS has a significant lag phase[1], K_M and k_{cat} were determined by fitting the data to the implicit Michaelis-Menten equation (see methods, Figure 3, Supplementary Figure 2). We have repeated this experiment independently, two times. Source data are provided with this paper.

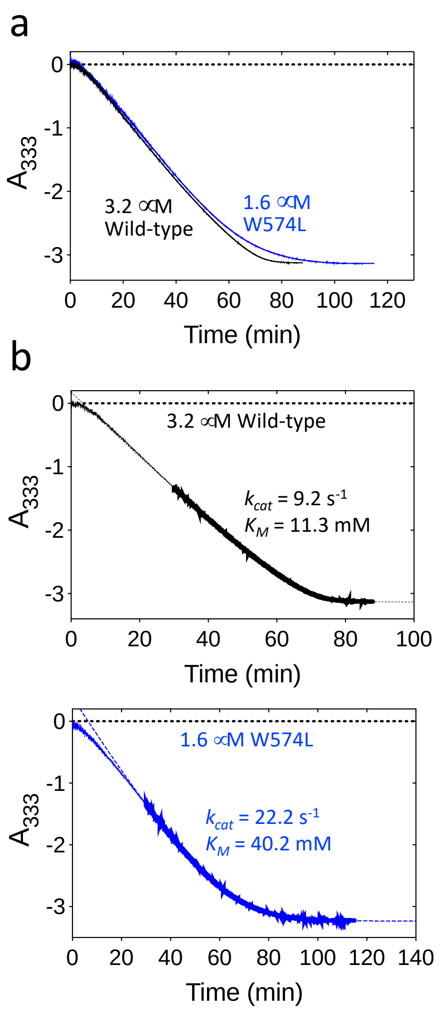

Supplementary Figure 2. Measurement of the kinetic parameters for WT *At*AHAS and the W574L mutant. a Progress curves for the reaction in the presence of 160 mM pyruvate, at 30° C. **b** Fitting of the progress curves with the implicit Michaelis Menten equation (Equation **5**, see Methods). For kinetic assessment, the curves were truncated, leaving only the thick section of the curve to be fitted, thereby removing the lag-phase. Source data are provided with this paper.

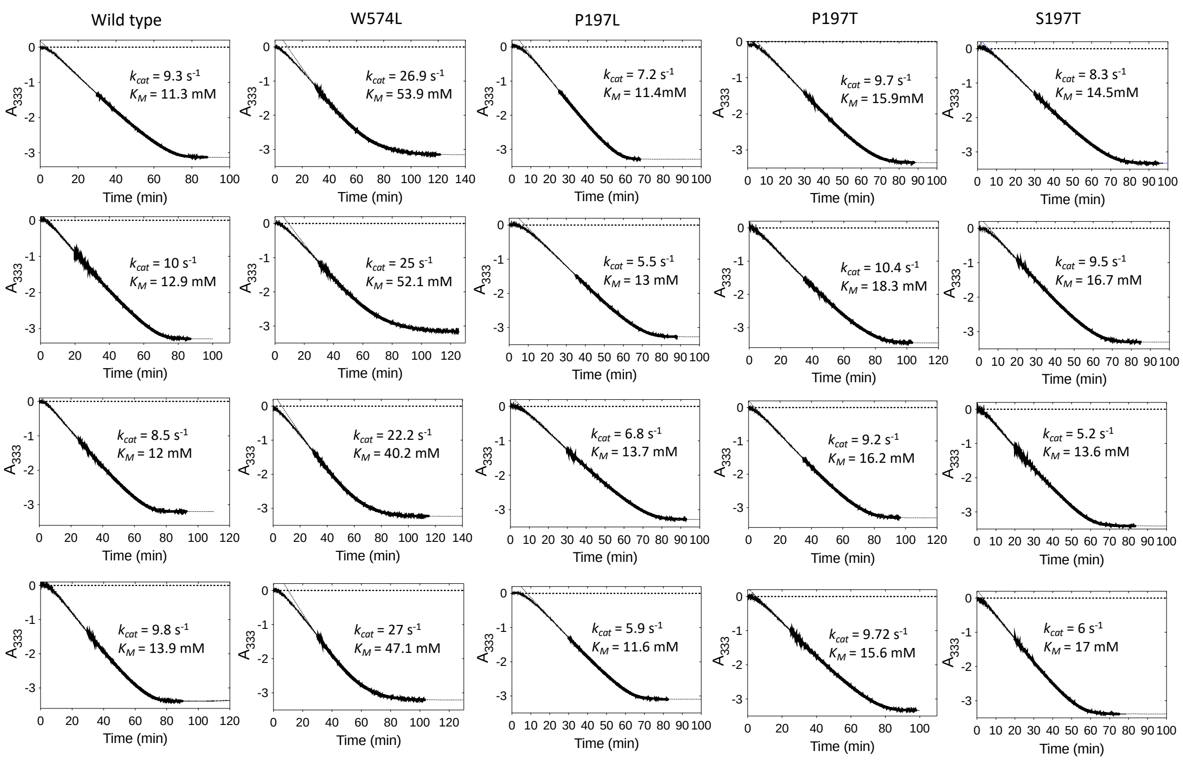

Supplementary Figure 3. Measurement, in quadruplicate, of the kinetic parameters of WT *At*AHAS and the mutants. The truncated progress curves using 160 mM pyruvate, at 30 °C, were fitted to the implicit Michaelis-Menten equation (Equation **5**, see Methods). Source data are provided with this paper.

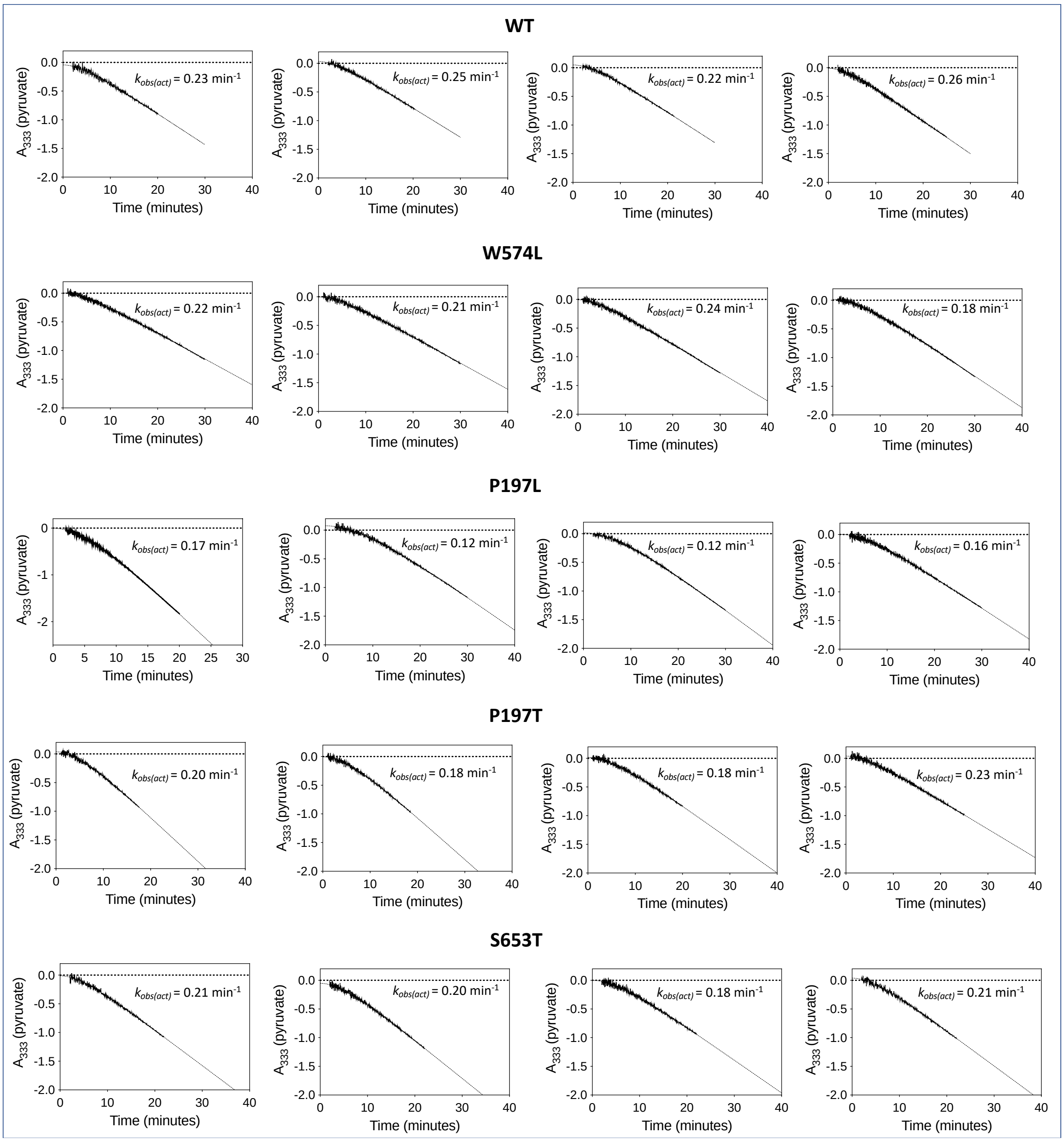

Supplementary Figure 4. Measurement, in quadruplicate, of the rate of activation ($k_{obs(act)}$) for WT *At*AHAS and the mutants. The progress curves corresponding to the lag phase of the AHAS reaction in the presence of 160 mM pyruvate, at 30° C, were fitted to Equation **6** (see Methods). Source data are provided with this paper.

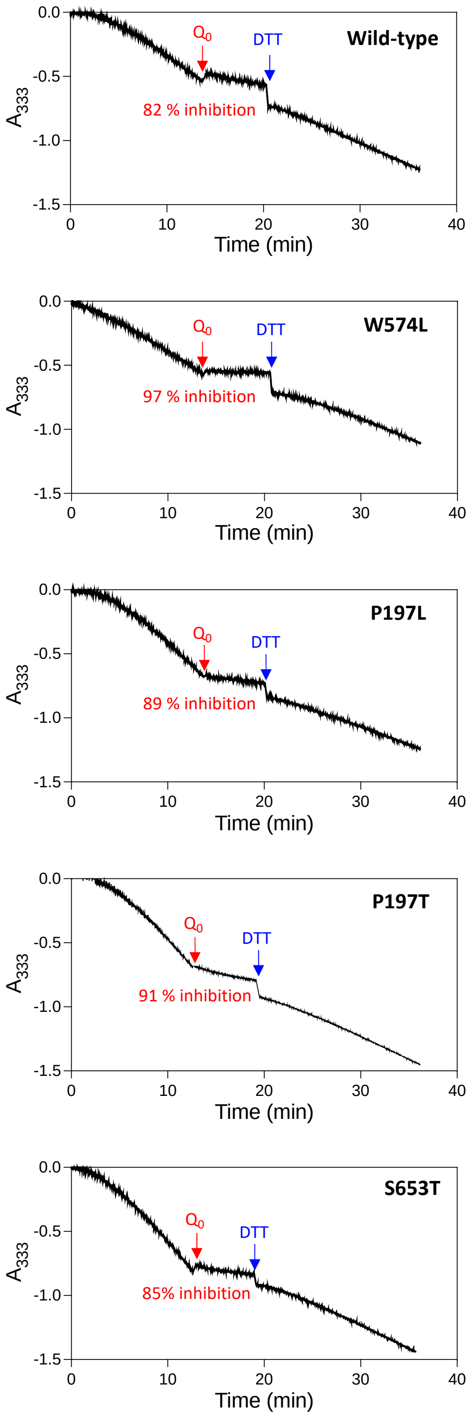

Supplementary Figure 5. Inhibition of WT *At*AHAS and the mutants by Q_0. *At*AHAS was incubated with 100 mM pyruvate at 30°C. After reaching full activation, the inhibition reaction was initiated by addition of 0.5 mM Q_0 to the sample cell. After seven minutes incubation with Q_0, 1 mM DTT was added to reduce the remaining Q_0, triggering re-activation of the enzyme. Source data are provided with this paper.

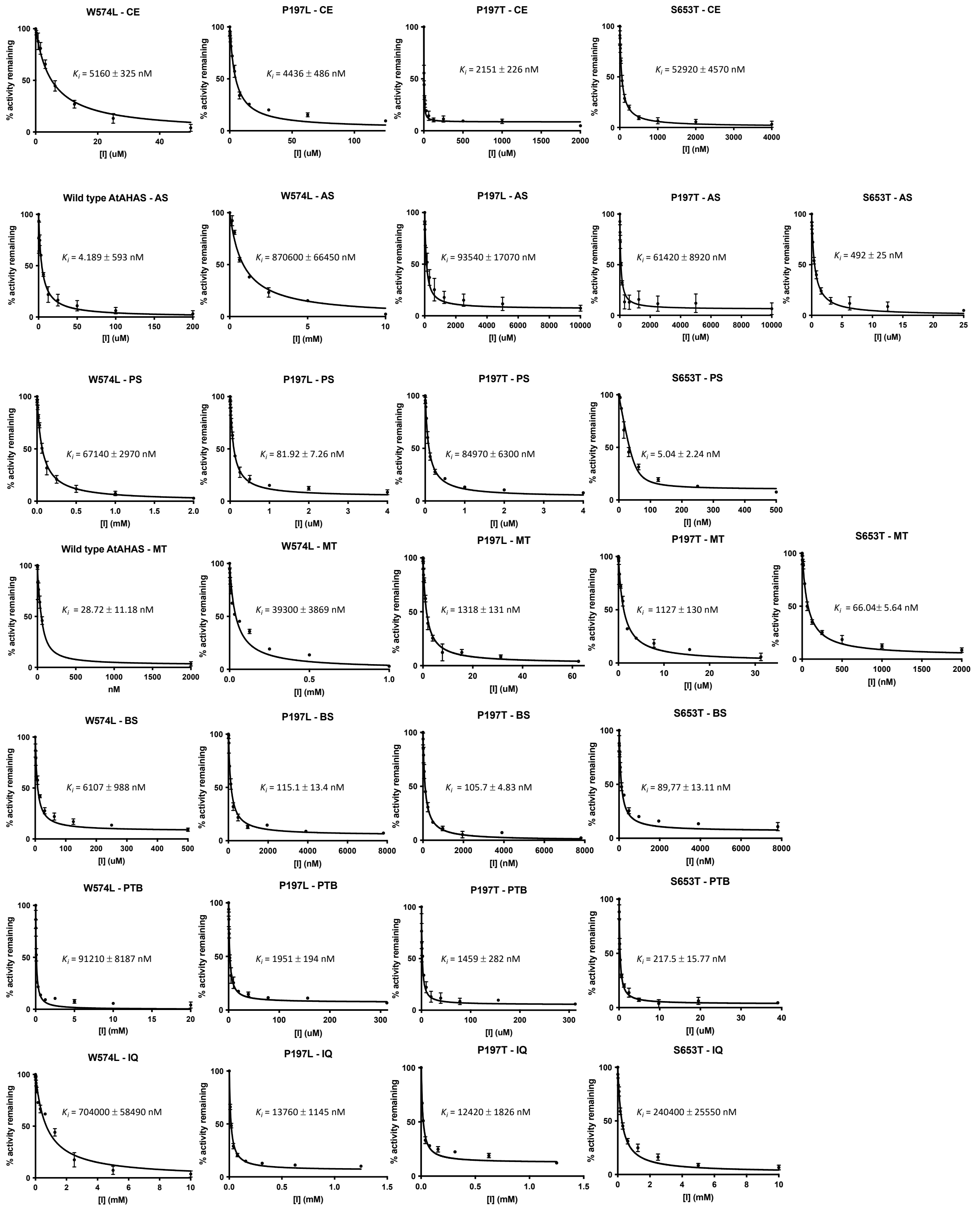

Supplementary Figure 6. Measurement of the K_i values for WT *At*AHAS and the mutants. The error bars represent the standard error of the mean of two independent replicates. [I] = inhibitor concentration. Source data are provided with this paper.

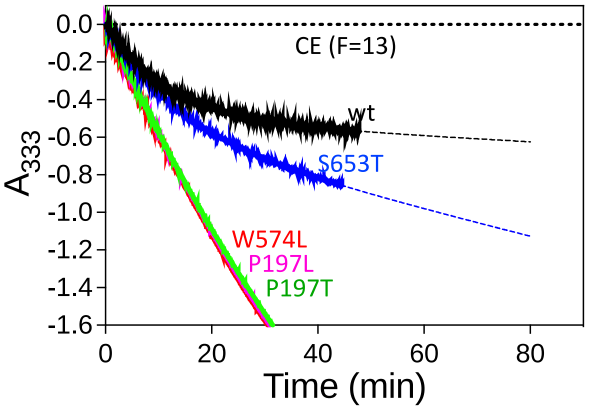

Supplementary Figure 7. Progress curves for the accumulative inhibition of WT *At*AHAS and its mutants by CE. F represents the ratio of the concentrations of the enzyme and CE in the assays (see Methods). For the W574L, P197L and P197T mutants, accumulative inhibition could not be detected. Source data are provided with this paper.

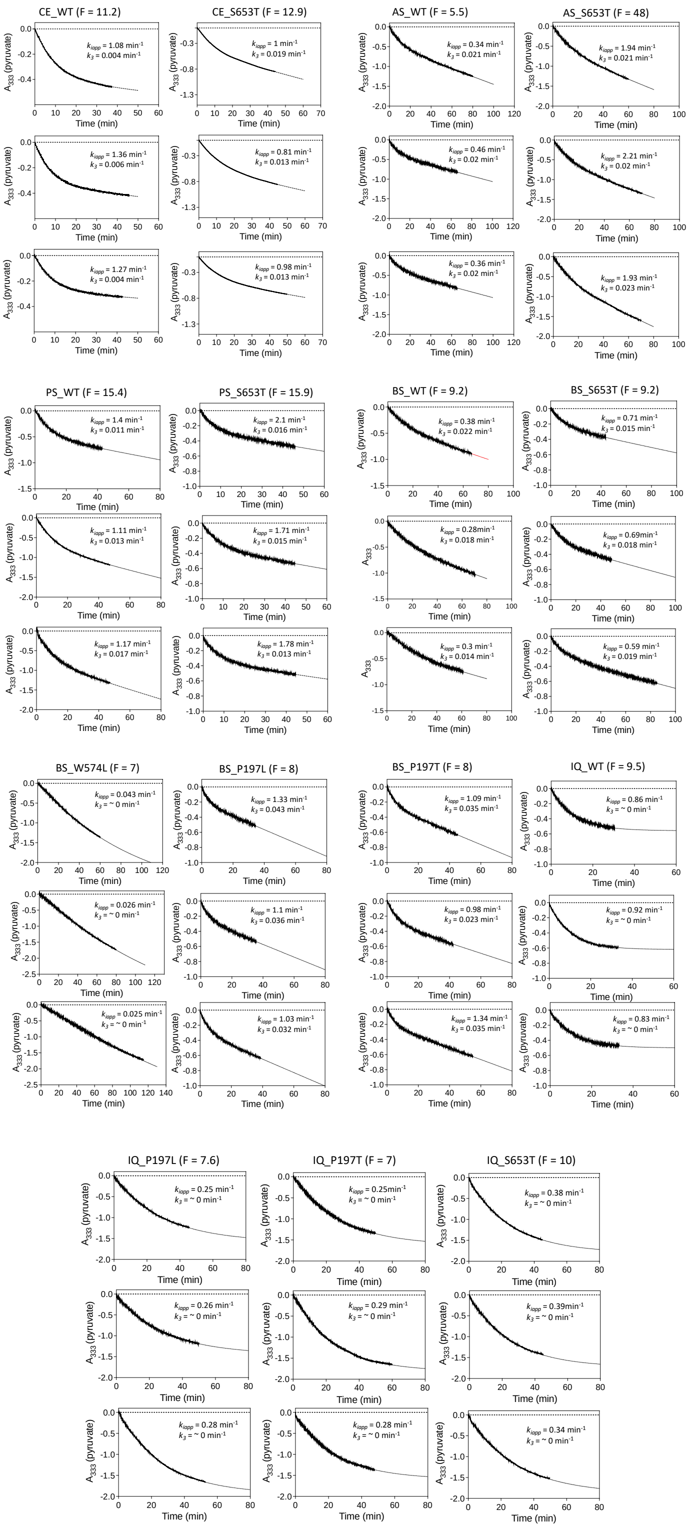

Supplementary Figure 8. Measurement of the rate constants of accumulative inhibition for WT *At*AHAS and its mutants. The progress curves were fitted to the equation of accumulative inhibition (Equation **7** [2]). The rate constants of enzyme inactivation (k_{iapp}) and enzyme recovery (k_3) were measured in triplicate. Source data are provided with this paper.

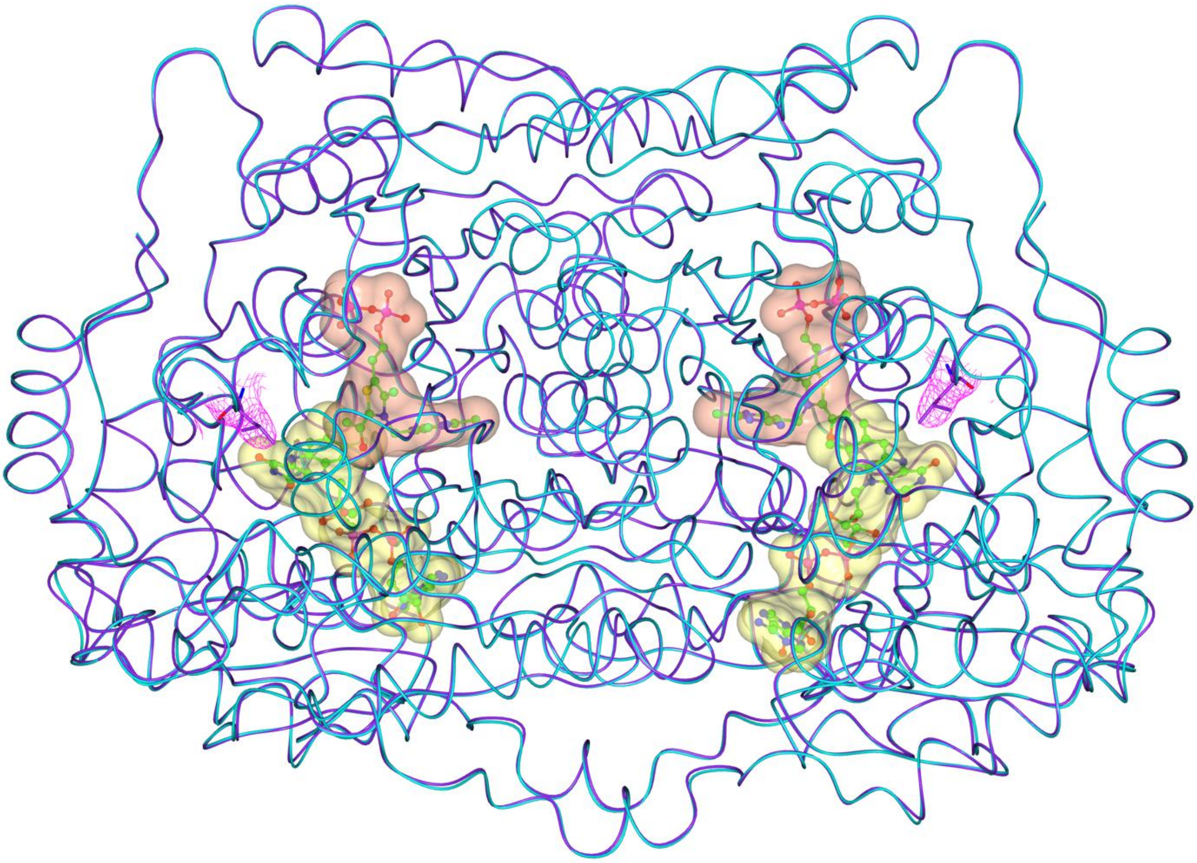

Supplementary Figure 9. Superimposition of the backbone of the WT and W574L *At*AHAS structures. The coordinates for the WT enzyme (cyan) were determined previously by Garcia *et al.* [3] and have the PDB code of 1YBH. The W574L *At*AHAS mutant is in purple. $2F_o$-F_c electron density for the W574L mutant is in pink mesh, contoured at 1.0 σ. FAD and ThDP are shown as ball and stick models with a transparent surface, in yellow and light brown, respectively.

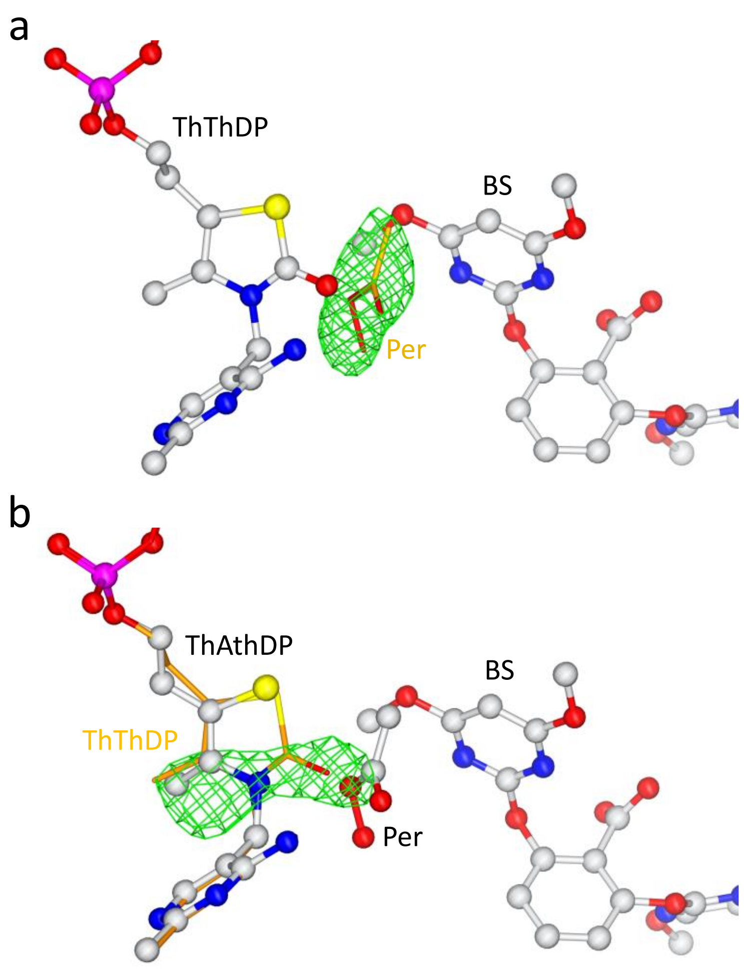

Supplementary Figure 10. **Electron density maps for ThDP in the W574L-BS complex.** Difference electron density maps for ThDP in the W574L-BS structure refined in presence of (a) ThThDP or in presence of (b) ThAthDP and peracetate (per). **a** When the structure of W574L-BS is refined with ThThDP (white, ball and stick), F_o-F_c omit electron density (green, 3.5 σ) remains in which a free peracetate (orange bonds) can fit. **b** Conversely, when the structure is refined with ThAthDP, F_o-F_c omit electron density (green, 3.5 σ) remains at the region where the oxygen of ThThDP would be located.

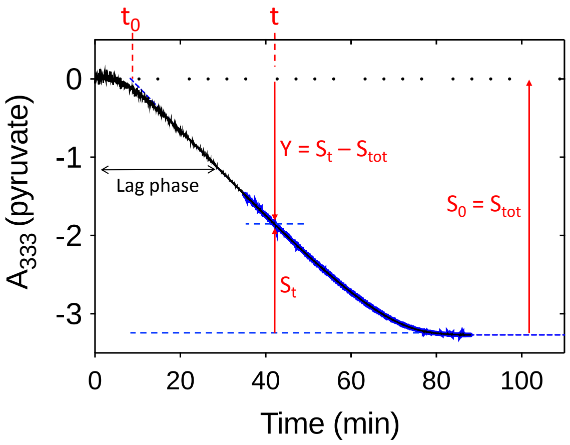

Supplementary Figure 11. Example of a curve used for the calculation of K_M and k_{cat}. The blue region of the curve represents the data points that were selected for the fitting to the implicit Michaelis-Menten equation, corresponding to the reaction after AHAS has been fully activated (data points belonging to the lag-phase (in black) have been removed).

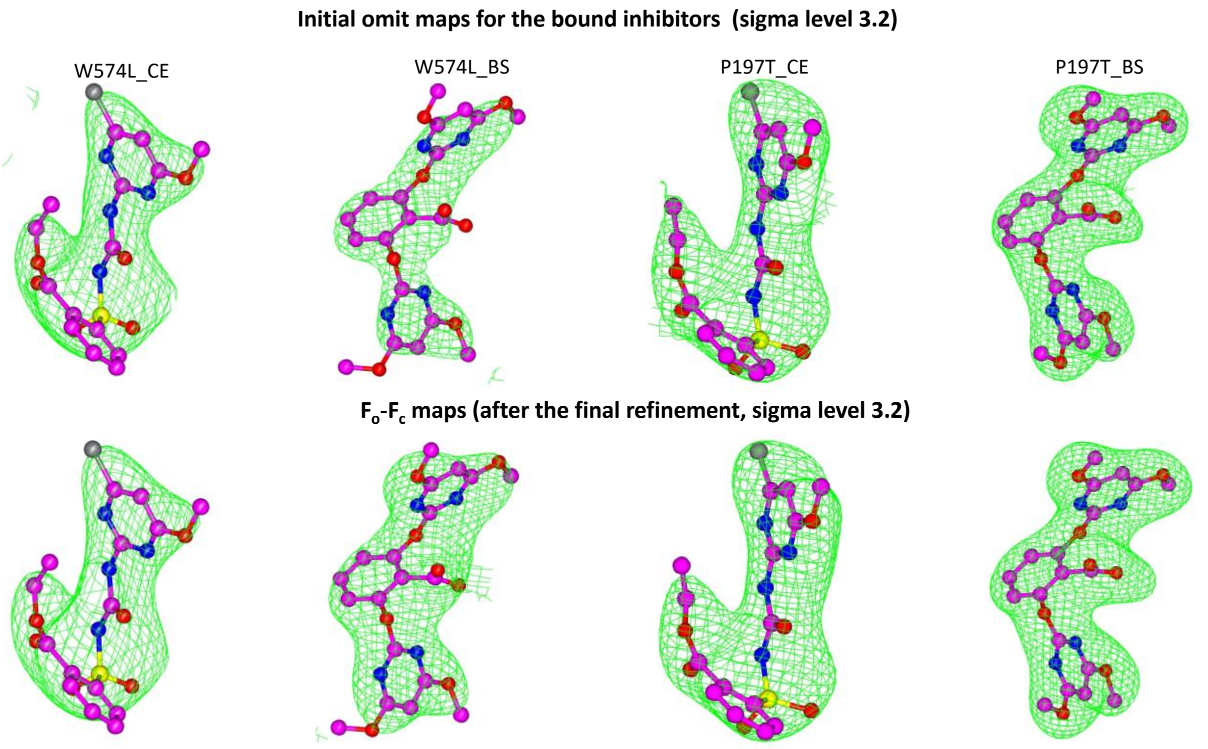

Supplementary Figure 12. **F_o-F_c omit electron density maps.** The unbiased F_o-F_c omit maps (green mesh) were created immediately after molecular replacement and prior to adding the herbicides to the refinement. The F_o-F_c maps (green mesh) without inhibitors were created using the final map but with the herbicides omitted.

Supplementary Table 1. **Resistance mutations observed in weed AHASs [4].**

**Amino acid**	**Number**	**Mutation**	**Herbicide class most affected**	**Weed family**
P197	92	T, H, R, L, Q, S, A, I	All five	*Asteraceae, Chenopodiaceae, Amaranthaceae, Scrophularlaceae, Papaveraceae, Cyperaceae, Poaceae, Brassicaceae, Pontederiaceae, Caryophyllaceae, Alismataceae, Lamiaceae, Rubiaceae*
W574	56	L, G, M, R	All five	*Asteraceae, Amaranthaceae, Chenopodiaceae, Brassicaceae, Cyperaceae, Poaceae, Caryophyllaceae, Papaveraceae, Rubiaceae, Polygonaceae, Euphorbiaceae, Boraginaceae*
S653	14	N, T, I	PYBs, IMIs, SCTs	*Amaranthaceae, Poaceae, Rubiaceae*
D376	13	E	All five	*Chenopodiaceae, Pontederiaceae, Amaranthaceae, Asteraceae, Rubiaceae, Brassicaceae, Cyperaceae, Poaceae*
A122	12	T, V, Y, S, N	TPs, SUs, PYBs, IMIs	*Asteraceae, Solanaceae, Amaranthaceae, Poaceae, Brassicaceae*
A205	6	V, F	All five	*Asteraceae, Amaranthaceae, Solanaceae, Poaceae*
G654	2	E, D	IMIs	*Poaceae*
R377	1	H	All five	Poaceae

*These data were obtained from the International herbicide resistance weed data base curated by Dr Ian Heap [4]. Source data are provided with this paper.

Supplementary Table 2. K_M and k_{cat} values for WT *At*AHAS and the mutants.

	WT	W574L	P197L	P197T	S653T
k_{cat} (s^{-1})	9.4 ± 0.5	25.0 ± 2.0	6.3 ± 0.8	9.8 ± 0.5	7.3 ± 2.2
K_M (mM)	12.5 ± 0.6	48.3 ± 3.1	12.4 ± 0.6	16.5 ± 1.3	15.5 ± 0.8
k_{cat}/K_M ($s^{-1}mM^{-1}$)	0.75 ± 0.10	0.52 ± 0.17	0.50 ± 0.16	0.59 ± 0.16	0.47 ± 0.25

Numbers before and after "±" represent mean and standard error of the four replicates. Source data are provided with this paper.

Supplementary Table 3. Rate of activation for WT *At*AHAS and the mutants and % inhibition of AHAS activity by 0.5 mM Q_0.

	**WT**	**W574L**	**P197L**	**P197T**	**S653T**
$k_{obs(act)}$ (min^{-1})	0.24 ± 0.01	0.21 ± 0.01	0.14 ± 0.01	0.19 ± 0.02	0.20 ± 0.02
% inhibition by 0.5 mM Q_0	82	97	89	91	85

Numbers before and after "±" represent mean and standard error of the four measurements. Source data are provided with this paper.

Supplementary Table 4. Field isolate resistance data for the mutations discussed in this study.

			PTB	IMI	SU	TP	Year
**Trp 574**							
	Leu	*Xanthium strumarium*	R	R	R	R	1995
	Leu	*Amaranthus tuberculatus (=A. rudis)*	ND	R	R	R	1996
	Leu	*Amaranthus hybridus (syn: quitensis)*	ND	R	R	R	1997
	Leu	*Kochia scoparia*	ND	R	R	ND	1999
	Leu	*Sisymbrium orientale*	ND	R	R	R	1999
	Leu	*Ambrosia artemisiifolia*	ND	R	R	R	2001
	Leu	*Ambrosia trifida*	ND	R	R	R	2002
	Leu	*Raphanus raphanistrum*	ND	ND	ND	ND	2002
	Leu	*Amaranthus blitoides*	R	R	R	R	2003
	Leu	*Camelina microcarpa*	ND	R	R	R	2004
	Leu	*Amaranthus retroflexus*	ND	R	R	ND	2005
	Leu	*Amaranthus powellii*	ND	R	R	ND	2005
	Leu	*Sinapis arvensis*	ND	R	R	R	2005
	Leu	*Schoenoplectus juncoides*	R	R	R	ND	2007
	Leu	*Lolium rigidum*	ND	R	R	ND	2007
	Leu	*Alopecurus myosuroides*	ND	ND	R	ND	2008
	Leu	*Bidens subalternans*	R	R	R	R	2009
	Leu	*Stellaria media*	ND	ND	R	R	2010
	Leu	*Schoenoplectus mucronatus (=Scirpus mucronatus)*	R	R	R	R	2010
	Leu	*Salsola tragus*	ND	ND	R	ND	2010
	Leu	*Apera spica-venti*	ND	ND	R	R	2011
	Leu	*Papaver rhoeas*	ND	ND	R	ND	2011
	Leu	*Galium spurium*	ND	R	ND	R	2012
	Leu	*Polygonum convolvulus (=Fallopia convolvulus)*	ND	ND	R	R	2012
	Leu	*Echinochloa phyllopogon (=E. oryzicola)*	R	R	R	R	2013
	Leu	*Poa annua*	R	R	R	ND	2013
	Leu	*Echinochloa crus-galli var. crus-galli*	R	R	R	R	2013
	Leu	*Alopecurus aequalis*	ND	R	R	R	2015
	Leu	*Conyza canadensis*	R	R	R	ND	2015
	Leu	*Cyperus esculentus*	R	R	R	R	2015
	Leu	*Cyperus iria*	R	R	R	R	2015
	Leu	*Sorghum halepense*	ND	ND	R	ND	2015
	Leu	*Raphanus sativus*	R	R	R	R	2016
	Leu	*Amaranthus palmeri*	R	R	R	R	2016
	Leu	*Chamaesyce maculata*	ND	ND	R	ND	2016
	Leu	*Descurainia sophia*	R	R	R	R	2016
	Leu	*Galium aparine*	r	r	r	r	2019
	Leu	*Lithospermum arvense*	R	R	R	R	2019
**Pro 197**							
	Thr	*Kochia scoparia*	ND	S	R	R	1990
	Leu	*Kochia scoparia*	ND	ND	R	ND	1995
	Leu	*Amaranthus retroflexus*	R	R	R	R	2001
	Thr	*Raphanus raphanistrum*	ND	S	R	R	2002
	Thr	*Papaver rhoeas*	r	r	R	r	2004
	Thr	*Chrysanthemum coronarium*	R	r	R	r	2004
	Leu	*Helianthus annuus*	ND	ND	R	ND	2004
	Thr	*Lactuca serriola*	ND	r	R	r	2006
	Leu	*Schoenoplectus juncoides*	S	S	R	ND	2007
	Leu	*Thlaspi arvense*	ND	r	R	S	2007
	Thr	*Alopecurus myosuroides*	ND	ND	R	ND	2008
	Leu	*Lolium rigidum*	ND	S	R	ND	2008
	Leu	*Descurainia sophia*	ND	ND	R	ND	2008
	Leu	*Papaver rhoeas*	ND	R	R	S	2009
	Thr	*Apera spica-venti*	ND	ND	R	r	2011
	Leu	*Anthemis cotula*	ND	r	R	r	2011
	Thr	*Anthemis cotula*	ND	r	R	r	2011
	Leu	*Sonchus asper*	ND	R	R	ND	2012
	Thr	*Capsella bursa-pastoris*	ND	ND	R	ND	2012
	Leu	*Capsella bursa-pastoris*	ND	ND	R	ND	2012
	Thr	*Hordeum murinum ssp. leporinum*	ND	r	R	ND	2012
	Thr	*Schoenoplectus juncoides*	ND	S	R	ND	2013
	Thr	*Alopecurus aequalis*	ND	r	r	r	2015
	Leu	*Senecio vulgaris*	ND	r	R	S	2016
	Leu	*Galium aparine*	r	r	R	r	2016
	Thr	*Galium aparine*	ND	ND	R	ND	2016
**Ser 653**							

	Thr	*Amaranthus powellii*	ND	R	S	ND	2001
	Thr	*Amaranthus tuberculatus (=A. rudis)*	ND	R	S	S	2001
	Thr	*Setaria viridis*	S	R	r	ND	2009
	Thr	*Avena fatua*	ND	R	ND	ND	2012
	Thr	*Amaranthus retroflexus*	ND	R	ND	ND	2015
	Thr	*Sorghum bicolor*	ND	R	S	ND	2017

S: Susceptible biotype; r: Moderate resistance (< 10-fold relative to sensitive biotype);

R: High Resistance (> 10-fold): ND: Not Determined.

PTB: Pyrimidinyl benzoates; IMI: Imidazolinones; SU: Sulfonylureas; TP: Triazolopyrimidine.

Supplementary Table 5. Data collection and refinement statistics for the *At*AHAS structures.

	W574L free (7U1U)	W574L-BS (7U25)	W574L-CE (7STQ)	P197T-CE (7U1D)	P197T-BS (7TZZ)
**Data collection**					
Space group	$P6_4\,2\,2$	$P6_4\,2\,2$	$P6_4\,2\,2$	$P6_4\,2\,2$	$P6_4\,2\,2$
Cell dimensions					
a=b, c (Å)	177.95, 184.73	179.95, 185.47	178.13, 184.76	178.48, 184.17	179.56, 184.84
$\alpha = \beta, \gamma$ (°)	90, 120	90, 120	90, 120	90, 120	90, 120
Resolution range (Å)	48.10-3.22	48.43-3.19	48.13-3.30	49.62-3.11	48.29-2.59
$^*R_{pim}$	0.044 (0.188)	0.047 (0.324)	0.044 (0.197)	0.034 (0.365)	0.016 (0.153)
<I>/< σI>	10.5 (2.3)	13.3 (2.0)	10.7 (2.6)	17.2 (1.6)	22.9 (3.1)
Completeness (%)	99.5 (97.5)	99.4 (96.4)	99.6 (98.7)	99.8(98.7)	99.6 (95.9)
**Refinement**					
Resolution range (Å)	48.10-3.22	48.43-3.19	48.13-3.30	49.62-3.11	44.89-2.59
No. reflection	28302	29776	26444	31668	54980
R_{work}	0.1856	0.1735	0.1781	0.1758	0.1546
R_{free}	0.2051	0.1924	0.2053	0.2048	0.1636
No. atoms					
Protein	4424	4456	4448	4468	4480
Ligand/ion	98	130	127	136	143
Water	N/A	N/A	N/A	N/A	284
β-factors (Å^2)					
Protein	76.84	83.78	83.75	81.48	63.03
Ligand/ion	75.83	78.41	82.38	82.57	60.70
Water	N/A	N/A	N/A	N/A	66.73
R.m.s.deviations					
Bond lengths (Å)	0.002	0.002	0.008	0.002	0.013
Bond angles (°)	0.494	0.482	0.736	0.484	1.427
**Validation**					
MolProbity score	1.33	1.27	1.46	1.12	1.18
Clashscore	3.79	2.64	5.08	3.28	3.06
Ramachandran favored (%)	97.05	96.55	96.88	98.09	98.27

Number in brackets represent values in the outer shell. CC = correlation coefficient.

$$^*R_{pim} = \sum_{hkl}\left[\frac{1}{[N(hkl)-1]}\right]^{1/2}\sum_i\left|I_i(hkl) - \left(I(hkl)\right)\right| / \sum_{hkl}\sum_i I_i(hkl)$$

, where $I_i(hkl)$ is the observed intensity and is the average intensity obtained from multiple observations of symmetry related reflections. Clashscore is defined as the number of overlaps ≥ 0.4 Å per thousand atoms. Hyperlinks for the pdb codes are //doi.org/10.2210/pdb7U1U/pdb, //doi.org/10.2210/pdb7U25/pdb, //doi.org/10.2210/pdb7STQ/pdb, //doi.org/10.2210/pdb7U1D/pdb, and //doi.org/10.2210/pdb7TZZ/pdb, respectively.

Supplementary Table 6. Primers used for *At*AHAS site-directed mutagenesis.

**Name**	**Primer sequence**
P197L-F	5’ ACA GGA CAA GTC CTT CGT CGT ATG ATT 3’
P197L-R	5’ AAT CAT ACG ACG AAG GAC TTG TCC TGT 3’
P197T-F	5’ ACA GGA CAA GTC ACT CGT CGT ATG ATT 3’
P197T-R	5’ AAT CAT ACG ACG AGT GAC TTG TCC TGT 3’
W574L-F	5’ ATG GTT ATG CAA TTG GAA GAT CGG TTC 3’
W574L-R	5’ GAA CCG ATC TTC CAA TTG CAT AAC CAT 3’
S653T-F	5’ CCG ATG ATC CCG ACT GGT GGC ACT TTC 3’
S653T-R	5’ GAA AGT GCC ACC AGT CGG GAT CAT CGG 3’

Supplementary references

[1] Lonhienne, T., Garcia, M. D. & Guddat, L. W. The role of a FAD cofactor in the regulation of acetohydroxyacid synthase by redox signaling molecules. *J Biol Chem.* **292**, 5101-5109 (2017).

[2] Lonhienne, T., *et al.* Commercial herbicides can trigger the oxidative inactivation of acetohydroxyacid synthase. *Angew Chem Int Ed Engl.* **55**, 4247-51 (2016).

[3] Garcia, M. D., Wang, J. G., Lonhienne, T. & Guddat, L. W. Crystal structure of plant acetohydroxyacid synthase, the target for several commercial herbicides. *FEBS J.* **284**, 2037-2051 (2017).

[4] Heap, I. The international herbicide-resistant weed database. https://www.weedscience.org/Home.aspx (2020).
