# Supplementary material for: Structural basis of resistance to herbicides that target acetohydroxyacid synthase
Source: Nat Commun. 2022 Jun 11;13:3368. doi: 10.1038/s41467-022-31023-x (PMC9188596; doi:10.1038/s41467-022-31023-x)
Supplement: Supplementary file 2 — Reporting Summary [file 41467_2022_31023_MOESM2_ESM.pdf]

nature portfolio

Corresponding author(s): Luke Guddat

Last updated by author(s): 2022/18/03

Reporting Summary

Nature Portfolio wishes to improve the reproducibility of the work that we publish. This form provides structure for consistency and transparency in reporting. For further information on Nature Portfolio policies, see our Editorial Policies and the Editorial Policy Checklist.

Statistics

For all statistical analyses, confirm that the following items are present in the figure legend, table legend, main text, or Methods section.

n/a	Confirmed	
X	☐	The exact sample size (*n*) for each experimental group/condition, given as a discrete number and unit of measurement
X	☐	A statement on whether measurements were taken from distinct samples or whether the same sample was measured repeatedly
X	☐	The statistical test(s) used AND whether they are one- or two-sided<br>*Only common tests should be described solely by name; describe more complex techniques in the Methods section.*
X	☐	A description of all covariates tested
X	☐	A description of any assumptions or corrections, such as tests of normality and adjustment for multiple comparisons
	X	A full description of the statistical parameters including central tendency (e.g. means) or other basic estimates (e.g. regression coefficient) AND variation (e.g. standard deviation) or associated estimates of uncertainty (e.g. confidence intervals)
X	☐	For null hypothesis testing, the test statistic (e.g. *F*, *t*, *r*) with confidence intervals, effect sizes, degrees of freedom and *P* value noted<br>*Give P values as exact values whenever suitable.*
X	☐	For Bayesian analysis, information on the choice of priors and Markov chain Monte Carlo settings
X	☐	For hierarchical and complex designs, identification of the appropriate level for tests and full reporting of outcomes
X	☐	Estimates of effect sizes (e.g. Cohen's *d*, Pearson's *r*), indicating how they were calculated

Our web collection on statistics for biologists contains articles on many of the points above.

Software and code

Policy information about availability of computer code

Data collection	For kinetics, the spectrophotometer from Shimadzu, UV-VIS 2600, was used. For X-ray data collection the MX1 beam line of the synchrotron of Melbourne (Australia) was used.
Data analysis	For kinetics, the data were analyzed and fitted to different equations using GraphPad Prism v9.3. For crystallographic data, the program Phenix was used to refine the structures. X-ray Data were processed using XDS, Phenix 1.9 (including Phaser). Model building was with COOT 0.8.9.

For manuscripts utilizing custom algorithms or software that are central to the research but not yet described in published literature, software must be made available to editors and reviewers. We strongly encourage code deposition in a community repository (e.g. GitHub). See the Nature Portfolio guidelines for submitting code & software for further information.

Data

Policy information about availability of data

All manuscripts must include a data availability statement. This statement should provide the following information, where applicable:

- Accession codes, unique identifiers, or web links for publicly available datasets
- A description of any restrictions on data availability
- For clinical datasets or third party data, please ensure that the statement adheres to our policy

Five structures have been determined, their ID codes at the Protein Data Bank (PDB) are 7TZZ, 7U1D, 7U25, 7STQ, and 7U1U. The structures will be accessible from PDB upon publication of the research. All other source data are available with the manuscript in the "source data" zip file.

Field-specific reporting

Please select the one below that is the best fit for your research. If you are not sure, read the appropriate sections before making your selection.

☒ Life sciences ☐ Behavioural & social sciences ☐ Ecological, evolutionary & environmental sciences

For a reference copy of the document with all sections, see nature.com/documents/nr-reporting-summary-flat.pdf

Life sciences study design

All studies must disclose on these points even when the disclosure is negative.

Sample size

Each kinetic experiment was performed as a continuous assay with measurements every 0.1s over 40-120 minutes, depending on the sample. Experiments were performed multiple times (duplicate, triplicate or quadruplicate) see below. For the Ki measurements each value was calculated based on 12 absorbance values in two runs. Each set of 12 are linked by the curve fitting analysis by Prism. This is a standard procedure employed in all enzymology laboratories. For the lag phase and accumulative inhibition measurements we have performed 3 replicates. For Km and Kcat values, lag phase, and Qo inhibition we performed these experiments in quadruplicate which allowed us to calculate standard error of the mean. Quadruplicates were used in this experiment. However, triplicate measurements would have been sufficient as each data point is internally consistent with the others.

Data exclusions

Replication

All attempts at replication were successful. No data were excluded.

Kinetic assays were performed in triplicate or quadruplicate on separate days

Randomization

The kinetic experiments were not randomized. This is never performed for this type of study. In the initial stages there is a trial and error to determine the approximate range of the parameters for the experiment. Then we are able to determine the optimal values for enzyme concentration, substrate concentration, temperature, buffer. Without this information we cannot proceed to carry out the experiment, and thus cannot be blind.

Blinding

Investigators were aware of which samples were being tested (ie the different mutants and compounds). It would not have been possible to link the assay results to the plots without making mistakes. The sample tubes are all labelled with mutant name or compound name.

Reporting for specific materials, systems and methods

We require information from authors about some types of materials, experimental systems and methods used in many studies. Here, indicate whether each material, system or method listed is relevant to your study. If you are not sure if a list item applies to your research, read the appropriate section before selecting a response.

Materials & experimental systems

n/a	Involved in the study	
☒	☐	Antibodies
☒	☐	Eukaryotic cell lines
☒	☐	Palaeontology and archaeology
☒	☐	Animals and other organisms
☒	☐	Human research participants
☒	☐	Clinical data
☒	☐	Dual use research of concern

Methods

n/a	Involved in the study	
☒	☐	ChIP-seq
☒	☐	Flow cytometry
☒	☐	MRI-based neuroimaging
