# Supplementary figures and images for: Structural basis of resistance to herbicides that target acetohydroxyacid synthase
Source: Nat Commun. 2022 Jun 11;13:3368. doi: 10.1038/s41467-022-31023-x (PMC9188596; doi:10.1038/s41467-022-31023-x)

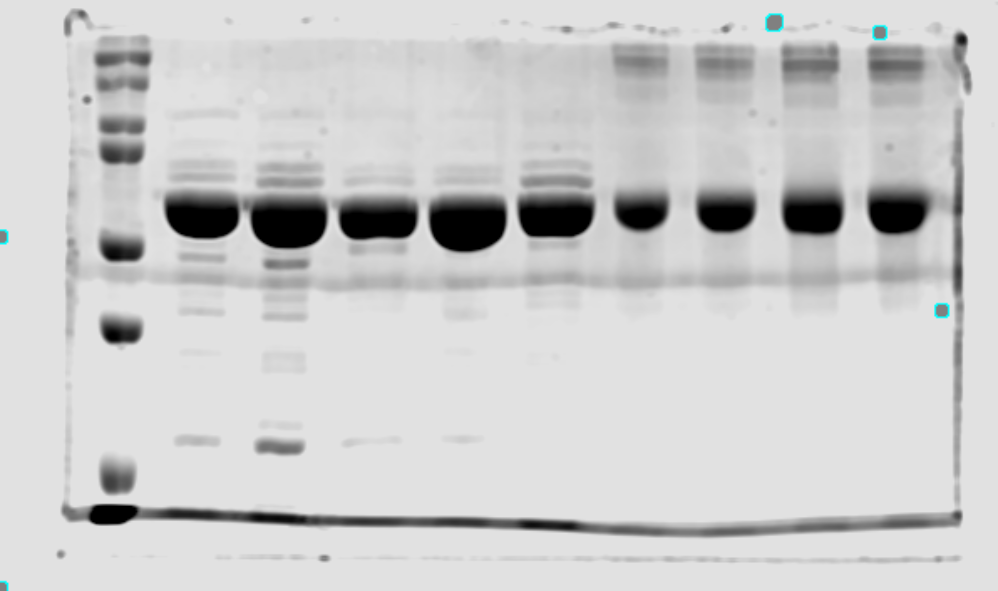

Supplement: Supplementary file 3 — Source Data [file 41467_2022_31023_MOESM3_ESM.zip › Raw data/Raw image of Supplementary Figure 1.png]
